# H1‐0 is a specific mediator of the repressive ETV6::RUNX1 transcriptional landscape in preleukemia and B cell acute lymphoblastic leukemia

**DOI:** 10.1002/hem3.70116

**Published:** 2025-04-02

**Authors:** Vera H. Jepsen, Andrea Hanel, Daniel Picard, Rigveda Bhave, Rebecca Hasselmann, Juha Mehtonen, Julian Schliehe‐Diecks, Carla‐Johanna Kath, Vithusan Suppiyar, Yash Prasad, Katerina Schaal, Jia‐Wey Tu, Nadine Rüchel, Ersen Kameri, Nan Qin, Herui Wang, Zhengping Zhuang, Rabea Wagener, Lena Blümel, Tobias Lautwein, Daniel Hein, David Koppstein, Gesine Kögler, Marc Remke, Sanil Bhatia, Merja Heinäniemi, Arndt Borkhardt, Ute Fischer

**Affiliations:** ^1^ Department of Pediatric Oncology, Hematology and Clinical Immunology, Medical Faculty Heinrich Heine University Düsseldorf Germany; ^2^ German Cancer Consortium (DKTK), Partner Site Essen/Düsseldorf Düsseldorf Germany; ^3^ Center for Integrated Oncology Aachen Bonn Cologne Düsseldorf (CIO ABCD) Bonn Germany; ^4^ Institute of Biomedicine, School of Medicine University of Eastern Finland Kuopio Finland; ^5^ Medical Faculty, Institute of Neuropathology Heinrich Heine University Düsseldorf Germany; ^6^ Medical Faculty, Institute for Transplantation Diagnostics and Cell Therapeutics Heinrich Heine University Düsseldorf Germany; ^7^ German Cancer Research Center (DKFZ) Heidelberg Germany; ^8^ Spatial and Functional Screening Core facility (SFS‐CF), Medical Faculty Heinrich Heine University Düsseldorf Germany; ^9^ Neuro‐Oncology Branch, Center for Cancer Research National Cancer Institute, NIH Bethesda Maryland USA; ^10^ Genomics Transcriptomics Laboratory, Biomedical Research Center Heinrich Heine University Düsseldorf Germany

## Abstract

*ETV6::RUNX1*, the most common oncogenic fusion in pediatric B cell precursor acute lymphoblastic leukemia (BCP‐ALL), induces a clinically silent preleukemic state that can persist in carriers for over a decade and may progress to overt leukemia upon acquisition of secondary lesions. The mechanisms contributing to quiescence of *ETV6::RUNX1*+ preleukemic cells still remain elusive. In this study, we identify linker histone H1‐0 as a critical mediator of the *ETV6::RUNX1*+ preleukemic state by employing human ‐induced pluripotent stem cell (hiPSC) models engineered by using CRISPR/Cas9 gene editing. Global gene expression analysis revealed upregulation of *H1‐0* in *ETV6::RUNX1*+ hiPSCs that was preserved upon hematopoietic differentiation. Moreover, whole transcriptome data of 1,727 leukemia patient samples showed significantly elevated *H1‐0* levels in *ETV6::RUNX1*+ BCP‐ALL compared to other leukemia entities. Using dual‐luciferase promoter assays, we show that ETV6::RUNX1 induces *H1‐0* promoter activity. We further demonstrate that depletion of H1‐0 specifically inhibits ETV6::RUNX1 signature genes, including *RAG1* and *EPOR*. Single‐cell sequencing showed that *H1‐0* is highly expressed in quiescent hematopoietic cells. Importantly, H1‐0 protein levels correspond to susceptibility of BCP‐ALL cells towards histone deacetylase inhibitors (HDACis) and combinatorial treatment using the H1‐0‐inducing HDACi Quisinostat showed promising synergism with established chemotherapeutic drugs. Taken together, our data identify H1‐0 as a key regulator of the *ETV6::RUNX1*+ transcriptome and indicate that the addition of Quisinostat may be beneficial to target non‐responsive or relapsing *ETV6::RUNX1*+ BCP‐ALL.

## Graphical abstract

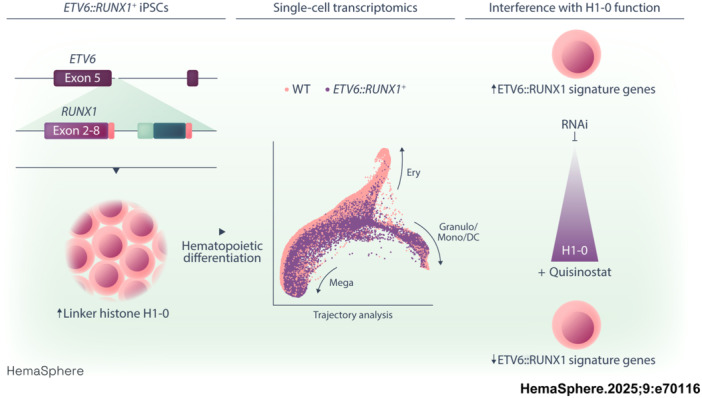

## INTRODUCTION

The chromosomal translocation t(12;21)(p13;q22) is the most common structural variation of pediatric B cell precursor acute lymphoblastic leukemia (BCP‐ALL) and results in the fusion of the two hematopoietic transcription factors ETS translocation variant 6 (*ETV6*) and runt‐related transcription factor 1 (*RUNX1*). The ETV6::RUNX1 fusion gene is acquired in utero in 1–5% of newborns^1^, ^2^ and requires further oncogenic mutations for progression to overt leukemia, predominantly including copy number alterations of genes involved in B cell development or cell cycle, e.g., *ETV6*, *PAX5*, *CDKN2A*, and *CDKN2B*.^3^, ^4^, ^5^, ^6^ Despite overall survival rates of *ETV6::RUNX1*+ pediatric leukemia exceeding 95% with current chemotherapy regimens, patients suffer from substantial acute and late toxicities, and disease recurrence is observed in approximately 5% of patients.^7^ This underlines the importance of understanding *ETV6::RUNX1*+ BCP‐ALL pathophysiology to enable further improvement of treatment.

While ETV6::RUNX1 itself is not sufficient for leukemic transformation, we and others have demonstrated that the fusion protein establishes a distinct preleukemic cell state.^8^, ^9^, ^10^, ^11^ ETV6::RUNX1 exerts an overall repressive effect on preleukemic cells, impeding early B cell differentiation, cell cycle, and inflammatory pathways, such as TGFβ signaling.^10^, ^11^, ^12^, ^13^ Transcriptional repression is conferred via the pointed domain (PNT) of the ETV6 moiety, while the runt‐homology domain (RHD) of the RUNX1 fusion part directly binds to promoters harboring the canonical RUNX1‐binding motif “TGYGGTY”.^14^, ^15^, ^16^, ^17^ ETV6::RUNX1 associates with multiple co‐repressors, including NCOR1, mSin3A, and histone deacetylases, such as HDAC3,^18^, ^19^ that induce changes in chromatin structure, leading to the characteristic repression of RUNX1 target genes.^20^

Modeling approaches of *ETV6::RUNX1*+ preleukemia and overt leukemia in mice were largely unable to reproduce restriction to B lineage leukemia seen in humans.^21^, ^22^, ^23^ This might be attributed to expression level‐dependent effects of ETV6::RUNX1, especially in models using viral transduction.^24^ Additionally, discrepancies between *ETV6::RUNX1*+ mouse and human models were linked to poor inter‐species conservation of GGAA repeat enhancers recently identified as key regulators of the *ETV6::RUNX1* + BCP‐ALL gene signature.^25^ Therefore, accurately recapitulating the intricate effects of ETV6::RUNX1 may necessitate modeling its function in a human background with physiological expression levels, as demonstrated by Böiers et al. using human‐induced pluripotent stem cells (hiPSCs).^10^

In this study, we detect consistent upregulation of linker histone H1‐0 in a preleukemic hiPSC model and leukemic blasts carrying the *ETV6::RUNX1* fusion gene. As a member of the H1 family of linker histones, H1‐0 affects chromatin compaction.^26^, ^27^ H1‐0 is heterogeneously expressed in solid tumors where it contributes to the intricate balance between cancer cell proliferation and differentiation.^27^ Our data reveal that H1‐0 regulates quiescence and acts as an important mediator of the ETV6::RUNX1 gene expression profile. Moreover, our study identifies the histone deacetylase inhibitor (HDACi) Quisinostat as a potential targeted approach for combinatorial drug treatment of *ETV6::RUNX1*+ leukemic cells.

## METHODS

### Cell lines and patient‐derived xenografts (PDX)

HW8 hiPSCs were generated from peripheral blood mononuclear cells (PBMCs) of a glioma patient using the CytoTune‐iPS 2.0 Sendai Reprogramming kit (Thermo Fisher Scientific, #A16517) following written informed consent. Study approval was obtained by the internal review board at the National Institutes of Health (NIH, protocol number: 16CN 069). Cellartis human iPSC line 12 (ChiPSC12, #Y00280) was purchased from Takara Bio. BCP‐ALL and 293T cell lines were obtained from the German Collection of Microorganisms and Cell Cultures (DSMZ). In‐house leukemia patient samples for injection into NSG mice (The Jackson Laboratory) were retrieved from the Biobank of the University Hospital of Düsseldorf following informed consent in accordance with the Declaration of Helsinki. Study approval was obtained by the ethics committee of the Medical Faculty of the Heinrich Heine University (study number: 2019‐566). All animal experiments adhered to regulatory guidelines set by the official committee at LANUV (Akt. 81‐02.04.2017.A441) and were authorized by the animal research institute (ZETT) at Heinrich Heine University Düsseldorf.

### Molecular cloning of a *RUNX1* HDR template

A *RUNX1* homology‐directed repair (HDR) template targeting *ETV6* intron 5 was constructed by combining *RUNX1* exon sequences 2–8 with a puromycin resistance gene under control of the human *EF‐1*α promoter. Homology arm sequences of ≈500 bp were polymerase chain reaction (PCR)‐amplified from ChiPSC12 genomic DNA and ligated to both sides of the HDR template. The *RUNX1* HDR template was subcloned into plasmid pUC19 (Addgene, #50005), linearized by PCR and concentrated by isopropanol precipitation to achieve a concentration ≥1 µg/µL. Single‐guide RNA (sgRNA) sequences targeting the 5′ region of *ETV6* intron 5 were designed using the online prediction tool CRISPOR (http://crispor.tefor.net/)^28^ and subcloned into the pUC19‐U6‐BbsI‐sgRNA plasmid. Target sequences used for CRISPR/Cas9 genome editing of hiPSCs were GGATGAGGCTAAATCCCTAA (hg38, chr12: 11,870,115–11,870,134, + strand) and GCCTAATTGGGAATGGTGCG (hg38, chr12: 11,870,054–11,870,073, − strand).

### CRISPR/Cas9 editing of hiPSCs

Following incubation with 10 µM Y‐27632 (STEMCELL Technologies, #72304) for 2 h, single‐cell suspensions of HW8 or ChiPSC12 hiPSCs were prepared using StemPro Accutase (Thermo Fisher Scientific, #A1110501). 10 × 10^6^ hiPSCs were resuspended in 100 µL P3 solution with supplement (Lonza, #V4XP‐3024) and transfected with 2.5 µg linearized *RUNX1* HDR template and 4 µg each of pCW‐Cas9 plasmid (Addgene, #50661) as well as two sgRNA plasmids using program CD‐118 of the 4D Nucleofector system (Lonza). hiPSCs were plated onto 10 cm Geltrex‐coated dishes in mTeSR Plus/Y‐27632. Medium was exchanged to mTeSR Plus without Y‐27632 after 24 h. Selection with 0.5 µg/mL puromycin (Thermo Fisher Scientific, #A1113803) was commenced 48 h after Nucleofection and single colonies were picked under microscopic guidance into a 96‐well plate at days 7–10. Clones were expanded for subsequent confirmation of correct HDR template insertion.

### In vitro differentiation of hiPSCs

Feeder‐free differentiation of hiPSCs was performed using the STEMdiff Hematopoietic kit (Stemcell Technologies, #05310) according to the manufacturer's instructions. Briefly, hiPSCs were seeded onto Geltrex‐coated (Thermo Fisher Scientific, #A1413301) 12‐well plates as aggregates. The next day (D0), medium A (containing bFGF, BMP4, and VEGFA) was added to wells containing 16–40 hiPSC colonies to induce mesodermal differentiation, and a half‐medium change was performed on D2. On D3, medium A was removed and medium B (containing bFGF, BMP4, VEGFA, SCF, Flt3L, and TPO) was added to induce hematopoietic differentiation. Half‐medium changes were performed on D5, D7, and D10. After 12 days, differentiated hematopoietic cells float in suspension and were harvested from the supernatant for downstream analyses.

### siRNA‐mediated H1‐0 knockdown

Specific siRNA sequences for knockdown of H1‐0 in REH cells were designed using the Eurofins siRNA design tool (https://eurofinsgenomics.eu/en/ecom/tools/sirna-design/) and purchased from Eurofins Genomics. Sequences are listed in Table S1. The 1 × 10^6^ REH cells were transfected with 200 pmol of each siRNA pool (siCtrl, siH1‐0_1, siH1‐0_2) in the 100 µL nucleocuvette format using the 4D Nucleofector system (Lonza, SF solution, program DS‐150).

### Dual‐luciferase reporter assay

Human *H1‐0* promoter sequence (nucleotides −351 to +161 from TSS) was PCR‐amplified from REH cell genomic DNA and inserted into Firefly luciferase vector pGL4.22 (Promega, #E6771) at KpnI and HindIII restriction sites. 293T cells at 50%–70% confluency were transfected with 755 ng plasmid DNA using Xfect Transfection Reagent (Clontech Laboratories, #631317) in 24‐well plates according to the manufacturer's instructions. To determine the effect of ETV6::RUNX1 on *H1‐0* promoter activation, each well was transfected with 500 ng pGL4.22 vector with or without *H1‐0* promoter expression as well as 5 ng Renilla luciferase control plasmid pGL4.73 (Promega, #E6911) and 250 ng of the respective pcDNA3.1 vectors (Thermo Fisher Scientific, #V79020) for expression of *ETV6::RUNX1* or *RUNX1* or empty vector in triplicates. To analyze the effect of Quisinostat on *H1‐0* promoter activation, transfection was performed with 500 ng pGL4.22 vector with *H1‐0* promoter expression and 5 ng Renilla luciferase control plasmid, and cells were treated with the indicated concentrations of Quisinostat or DMSO (1:10,000) for 24 h. Cells were lysed 48 h after transfection with Passive Lysis buffer and luciferase signal was measured on a Tecan SPARK 10 M reader using the Dual‐Luciferase Reporter Assay System (Promega, #E1910). Firefly luciferase signal was normalized to Renilla luciferase activity. Adequate protein expression of ETV6::RUNX1 and RUNX1 was determined by Western blot.

### In vitro inhibitor treatments

BCP‐ALL cell lines were treated with 1 µM JNJ‐26481585/Quisinostat at a concentration of 1 × 10^6^ cells per ml and RNA was extracted after 24 h for subsequent analysis of *H1‐0* expression by RT‐qPCR and RNA‐seq analysis. DMSO‐dissolved compounds were purchased from Selleck Chemicals and MedChem Express. For drug synergy analysis, Quisinostat (concentration range: 0.2–20 nM), AR‐42 (concentration range: 10–1000 nM), suberanilohydroxamic acid (SAHA)/Vorinostat (concentration range: 100–5000 nM), Vincristine (concentration range: 0.1–5 nM), Daunorubicin (concentration range: 1.5–50 nM), and Bortezomib (concentration range: 1–10 nM) were printed onto 384‐well plates (Corning, #3570) in a randomized fashion in increasing concentrations of 8 × 8 matrices using a D300e digital dispenser (Tecan) and normalized with DMSO (Sigma‐Aldrich, #2650). BCP‐ALL cell lines were seeded at a concentration of 200,000 cells/mL (6000 cells/well). Due to limited amount of cells, PDX samples and siRNA‐treated REH cells were seeded at 100,000 cells/mL (3000 cells/well). PDX samples were thawed and cultured for 24 h at 37°C and 5% CO_2_ in StemSpan SFEM II (Stemcell Technologies, #09605) with added StemSpan CD34+ expansion supplement (Stemcell Technologies, #02691) before performing drug screens. Plates were incubated for 72 h and viability was determined by CellTiter‐Glo Luminescent viability assay (Promega) using a Tecan SPARK 10 M reader. Most synergistic area scores (2 × 2 dose window) were determined using the zero interaction potency (ZIP) method using the SynergyFinder web application (version 3.0).^29^ A most synergistic area score above 5 was considered synergistic.

### Statistical analysis

Statistical analysis of data was performed using GraphPad Prism version 9.5.1. The number (*n*) of replicates and statistical tests are indicated in the figure descriptions. Statistical significance was considered for *p* values: *p < 0.05, **p < 0.01, and ***p < 0.001.

Additional methods can be found in the Supporting Information.

## RESULTS

### H1‐0 is upregulated in a preleukemic hiPSC model and BCP‐ALL expressing *ETV6::RUNX1*

**Figure 1:**
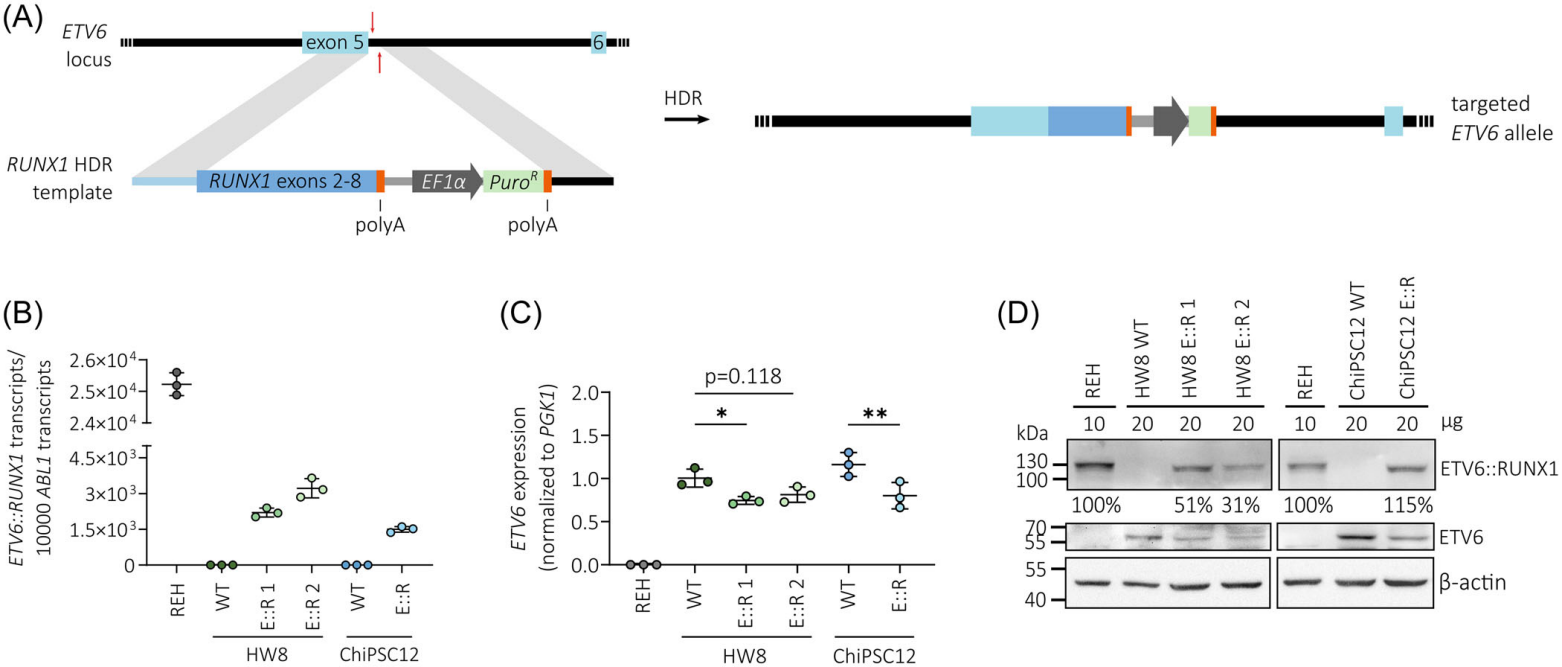
**Preleukemic** ***ETV6::RUNX1*** **+ hiPSC model**. **(A)** Scheme representing CRISPR/Cas9‐mediated targeted editing of the endogenous *ETV6* locus using a *RUNX1* homology‐directed repair (HDR) template. **(B)** Quantification of *ETV6::RUNX1* expression by RT‐qPCR in the *ETV6::RUNX1*+ cell line REH, HW8, and ChiPSC12 hiPSC lines. Data are presented as the mean ± standard deviation. **(C)** Relative *ETV6* expression determined by RT‐qPCR in REH and hiPSCs. Mean expression ± standard deviation is indicated and data were analyzed for statistical significance using an ordinary one‐way ANOVA (*p < 0.05, **p < 0.01). **(D)** Western blot analysis of REH and hiPSC lysates using antibodies directed against β‐actin, ETV6, and RUNX1 detecting the ETV6::RUNX1 (E::R) fusion protein.

To analyze specific gene expression patterns of *ETV6::RUNX1*‐translocated preleukemia in a model without additional secondary alterations, we generated monoclonal hiPSC lines derived from two donors: HW8 and ChiPSC12. We used a CRISPR/Cas9‐mediated knock‐in approach to directly fuse *RUNX1* exons 2–8 to *ETV6* exon 5 and to place the resulting fusion gene under the physiological control of the endogenous *ETV6* promoter (Figure 1A). We confirmed correct sequence of the *RUNX1* insert by genotyping PCR of the *ETV6* locus (Figure S1A,B) and Sanger sequencing (Figure S1C). ETV6::RUNX1 levels in the hiPSC lines as detected by reverse transcription quantitative PCR (RT‐qPCR) and Western blot were lower compared to the *ETV6::RUNX1*+ BCP‐ALL cell line REH (Figure 1B). All hiPSC lines maintained typical hiPSC microscopic morphology and expression of the pluripotency markers *SSEA‐4*, *DNMT3B*, *GDF3*, *POU5F1*, and *NANOG* as determined by flow cytometric analyses and RT‐qPCR; chromosomal integrity was confirmed by karyotype analysis (Figure S2). All *ETV6::RUNX1*+ hiPSC lines harbored a monoallelic insertion of the *RUNX1* HDR template at the *ETV6* locus as detected by PCR, RT‐qPCR, and Sanger sequencing (Figure S1D–G). Since the *RUNX1* HDR template disrupts one *ETV6* allele, expression of full‐length *ETV6* was lower in the CRISPR/Cas9‐edited hiPSCs compared to the wild‐type controls (Figure 1C,D). This aligns with the genetic profile of *ETV6::RUNX1*+ ALL patients who commonly exhibit heterozygosity for the fusion gene. Since *ETV6* exon 6 is not retained in the *ETV6::RUNX1* fusion gene, full‐length *ETV6* was detected using an RT‐qPCR spanning exons 5 and 6 (Figure S1F). REH cells served as negative control due to the deletion of the remaining copy of *ETV6* (Figure 1C,D).

**Figure 2:**
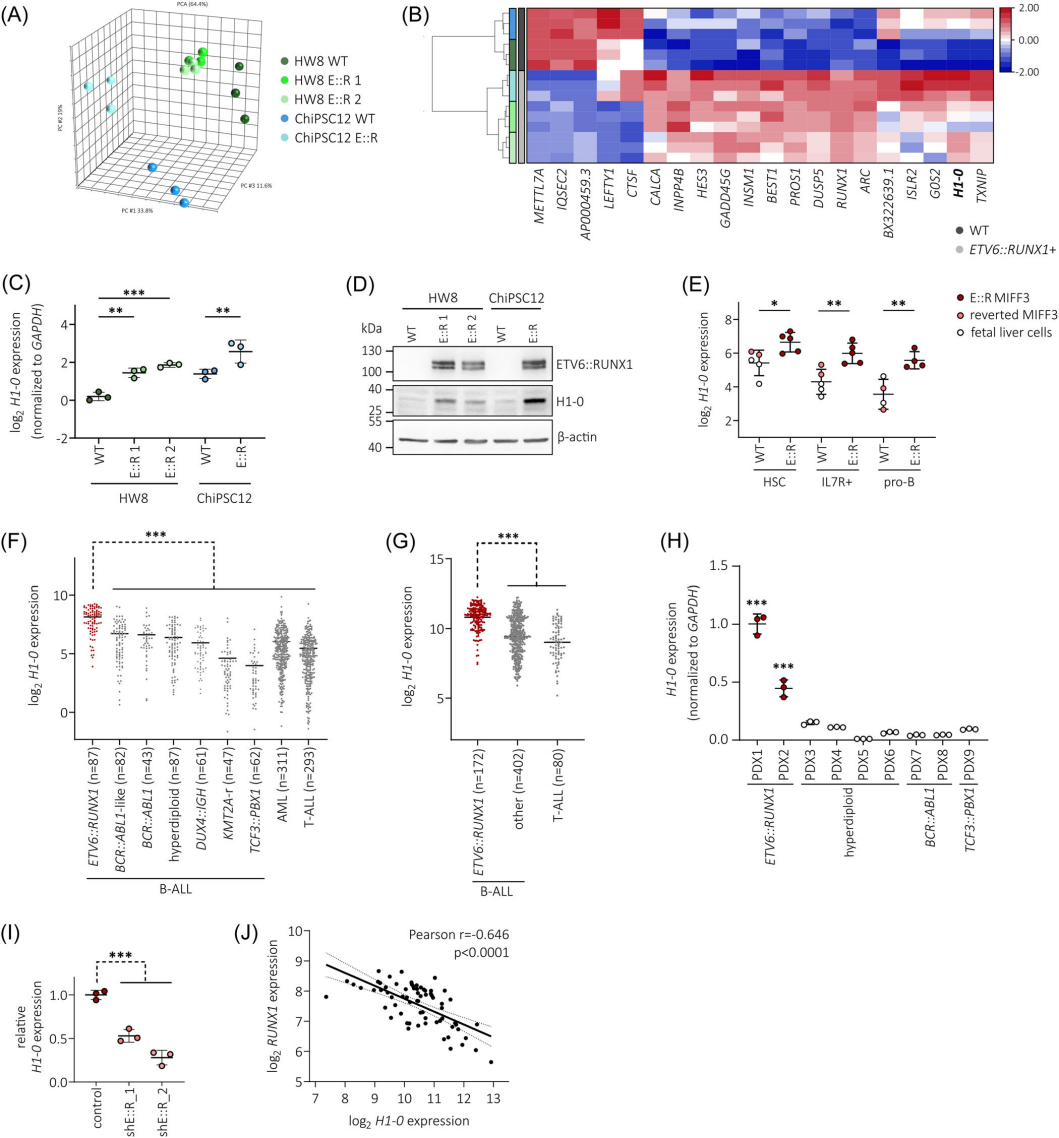
**H1‐0 is consistently upregulated in preleukemia and BCP‐ALL expressing** ***ETV6::RUNX1**.* **(A)** Principal component analysis (PCA) plot of *ETV6::RUNX1*+ (E::R) and wild‐type (WT) hiPSC transcriptome profiles based on all detected genes (*n* = 16,328). **(B)** Hierarchical clustering analysis of differentially expressed genes (absolute fold change > 2 and *p* < 0.05) between *ETV6::RUNX1*+ and WT hiPSCs detected by RNA‐seq. **(C)** *H1‐0* expression levels determined by RT‐qPCR in *ETV6::RUNX1*+ and WT hiPSCs subjected to RNA‐seq. Values were normalized to HW8 WT expression levels as well as to *GAPDH* expression. **(D)** Representative Western blot analysis of ETV6::RUNX1, H1‐0, ETV6, and β‐actin levels in *ETV6::RUNX1*+ and WT hiPSCs. **(E)** *H1‐0* levels in HSCs (CD19‐CD34+CD45RA‐), IL7R+ (CD19‐CD34+CD45RA+IL7R+), and pro‐B (CD19+CD34+) cells differentiated from *ETV6::RUNX1*+ or reverted MIFF3 hiPSCs, and fetal liver cells. Data are derived from an RNA‐seq dataset by Böiers et al. (accession number E‐MTAB‐6382^10^). Data were analyzed for statistical significance using an ordinary one‐way ANOVA (*p < 0.05, **p < 0.01). *H1‐0* levels across two leukemia patient cohorts derived from the **(F)** PeCan St. Jude database^30^, ^31^ and **(G)** an expression microarray dataset (accession number GSE87070^32^). The number of patients per leukemia entity and mean expression is indicated. Data were analyzed for statistical significance using an ordinary one‐way ANOVA (***p < 0.001). **(H)** *H1‐0* expression was quantified by RT‐qPCR in PDX samples (*n* = 9). Mean expression ± standard deviation is shown. **(I)** RNA‐seq expression levels of *H1‐0* in control and *ETV6* shRNA‐transduced REH cells. Data are derived from E‐MTAB‐10308^11^ and are normalized to control shRNA. Mean expression ± standard deviation is indicated. Statistical significance was determined by performing a one‐way ANOWA (***p < 0.001). **(J)** Pearson correlation of *H1‐0* and *RUNX1* expression in healthy bone marrow cells (*n* = 71) derived from the MILE study (R2 platform, accession number GSE13159^33^).

To identify significantly dysregulated genes in *ETV6::RUNX1*+ preleukemic cells, we performed bulk RNA‐seq of *ETV6::RUNX1*+ and wild‐type hiPSCs. Principal component analysis (PCA) clearly separated samples according to genotype (Figure 2A). Altogether, we found consistent differential expression of 20 genes with an absolute fold change > 2 and *p* < 0.05 in the three *ETV6::RUNX1*+ hiPSC lines compared to the respective wild‐type hiPSC lines (Figures 2B and S3 and Tables S2–S5). These genes remained significantly dysregulated using a harsher cut‐off of false discovery rate (FDR)‐adjusted *p* value (or *q* value) < 0.1 (Table S5). Among these genes, *H1‐0* has previously been identified as the most significantly upregulated gene in dormant leukemia stem cell‐like cells.^34^ As a linker histone, H1‐0 is involved in epigenetic regulation of chromatin and affects cellular differentiation states,^27^ making it a compelling candidate for further investigation. Elevated levels of *H1‐0* identified by RNA‐seq in the *ETV6::RUNX1*+ hiPSCs were confirmed both by RT‐qPCR (2.4‐fold increased mean expression; Figure 2C) and Western blot (Figure 2D). Moreover, upregulation of *H1‐0* in *ETV6::RUNX1*+ preleukemic cells is preserved during the differentiation of hiPSCs along the B lymphoid lineage in a published RNA‐seq dataset^10^ (Figure 2E).

We previously found *H1‐0* expression to be restricted to *ETV6::RUNX1*+ bone marrow blasts compared to peripheral blood CD19+ cells,^8^ indicating that H1‐0 upregulation is preserved upon leukemic transformation and highly specific for leukemic cells carrying the *ETV6::RUNX1* fusion gene. To confirm this finding, we analyzed transcriptomic data derived from two patient cohorts encompassing a total of 1727 leukemia patient samples (PeCan St. Jude cohort^30^, ^31^ and GSE87070^32^). Additionally, we determined *H1‐0* expression in nine BCP‐ALL PDX samples by RT‐qPCR (*n* = 2 *ETV6::RUNX1*+, *n* = 4 high‐hyperdiploid, *n* = 2 *BCR::ABL1*+, *n* = 1 *TCF3::PBX1*+). Notably, *ETV6::RUNX1*+ BCP‐ALL showed significantly elevated *H1‐0* levels compared to other leukemia entities (Figure 2F–H). Moreover, *H1‐0* was downregulated upon *ETV6::RUNX1* knockdown in published RNA‐seq data^11^ of REH cells (*p* < 0.001, Figure 2I). In line with these observations, *H1‐0* expression closely anti‐correlates with *RUNX1* expression (Pearson *r* = −0.646, *p* < 0.0001, Figure 2J) in healthy bone marrow cells derived from the MILE study.^33^ Altogether, these data support an association of the *ETV6::RUNX1* fusion gene and linker histone H1‐0 expression.

### ETV6::RUNX1 skews lineage commitment during early hematopoiesis

**Figure 3:**
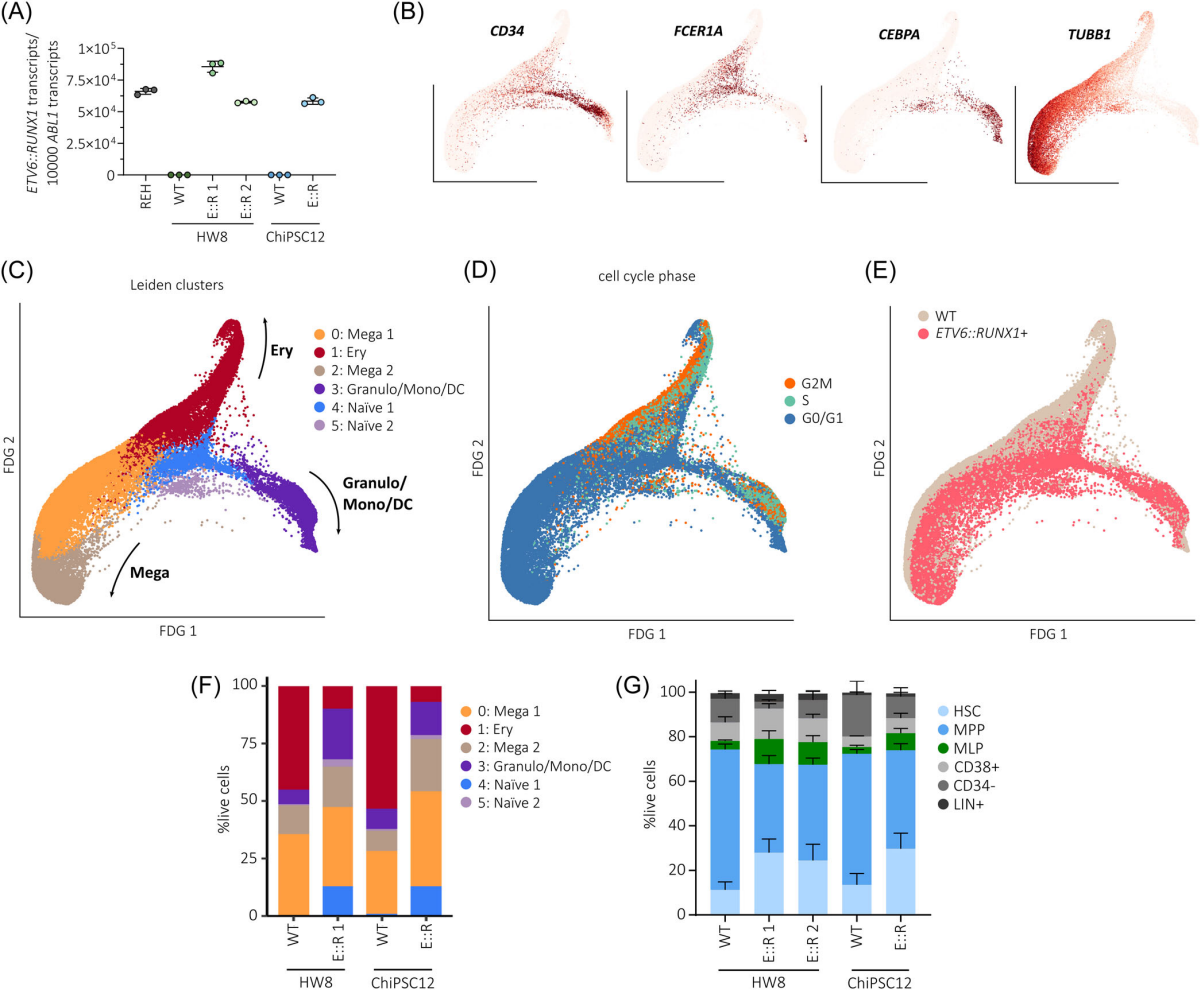
**ETV6::RUNX1 skews lineage‐commitment during early hematopoiesis**. **(A)** Quantification of *ETV6::RUNX1* expression by RT‐qPCR in REH and hiPSC‐derived HPCs. Mean expression ± standard deviation is indicated. Force‐directed graph (FDG) plots of HPC scRNA‐seq data derived from HW8 WT, HW8 E::R 1, ChiPSC12 WT, and ChiPSC12 E::R hiPSCs. Force‐directed graphs of **(B)** lineage‐defining genes (Naïve: *CD34*, *FCER1A*; Granulo/Mono/DC: *CEBPA*; Mega: *TUBB1*), **(C)** Leiden clusters, **(D)** cell cycle phase, and **(E)** genotypes. **(F)** Percentages of cell types defined by Leiden clustering in wild‐type and *ETV6::RUNX1*+ HPCs. **(G)** Frequencies of HSCs, MPPs, MLPs, CD38+, CD34−, and LIN+ cells in wild‐type or *ETV6::RUNX1*+ HPCs determined by flow cytometry. DC, dendritic cells; Ery, erythrocytes; Granulo, granulocytes; HSC, hematopoietic stem cells; Mega, megakaryocyte; MLP, multi‐lymphoid progenitors; Mono, monocytes; MPP, multipotent progenitors.

To delineate effects of ETV6::RUNX1 in hematopoietic cells, we performed in‐depth characterization of hematopoietic progenitor cells (HPCs) differentiated from *ETV6::RUNX1*+ and wild‐type hiPSCs. As confirmed by RT‐qPCR, *ETV6::RUNX1* expression in HPCs increased to levels comparable with REH cells (Figure 3A) and expression of common hiPSC pluripotency marker genes decreased during differentiation (Figure S4). To infer lineage‐commitment of hiPSC progenitors, we carried out single‐cell RNA‐seq (scRNA‐seq) and annotated our in vitro‐derived HPCs using published reference data (Figure S5A–C). Overlaying our data with published scRNA‐seq atlas data^35^ revealed that hiPSC‐derived HPCs clustered closer to fetal liver compared to fetal bone marrow or cord blood‐derived cells (Figure S5C). This finding is in keeping with other studies that showed similarity of hiPSC in vitro differentiation and early fetal hematopoiesis, for instance, in the fetal liver.^10^, ^36^ By mapping to fetal liver, fetal bone marrow and cord blood,^35^ as well as to adult bone marrow^37^ reference data, we identified three distinct trajectories of megakaryocyte, erythroid, and granulocyte/monocyte/dendritic cell (DC) progenitors in hiPSC‐derived cells (Figure S5A,B). Comparable differentiation trajectories were observed by using a diffusion map representation of the data, which showed a central node of naïve cells and three branches of lineage‐committed progenitors (Figure S5D). Of note, our data exhibited similar lineage commitment compared to a previous study that analyzed hiPSC‐derived hematopoietic progenitors generated through embryoid body differentiation.^36^ Expression of cell type‐specific marker genes confirmed commitment to megakaryocytes in Leiden clusters 0 and 2 (*TUBB1*, expression of additional marker genes shown in Figure S6), and commitment to granulocytes/monocytes/DCs in cluster 3 (*CEBPA*), while naïve progenitors in cluster 4 expressed *CD34* and the early megakaryocytic‐erythroid marker *FCER1A* (Figure 3B–C). Naïve progenitors in cluster 5 exhibited an intermediate expression profile of megakaryocyte and granulocyte/monocyte/DC marker genes. In line with previous work showing that megakaryocytic‐erythroid lineage specification is governed by cell cycle speed,^38^ we observed that erythroid‐committed cluster 1 was defined by differential expression of cell cycle regulators (Table S6 and Figure S5E), while megakaryocyte progenitors were annotated as non‐cycling (Figure 3D).

Classification of HPCs by genotype revealed that expression of *ETV6::RUNX1* skewed commitment towards granulocyte/monocyte/DC progenitors and resulted in increased abundance of naïve progenitors (Figure 3E,F; cell numbers per cluster are shown in Table S7). Comparison of *ETV6::RUNX1*+ and wild‐type cells within the annotated clusters identified the highest number of differentially expressed genes within the megakaryocyte‐ and erythrocyte‐committed clusters (0–2; Figure S5F and Table S8). In keeping with reduced commitment to erythrocyte progenitors, cell cycle scoring showed accumulation of non‐cycling G0/G1 cells in *ETV6::RUNX1*+ HPCs (FDR = 0.018, Figure S5G). Additionally, transcriptional diversity and activity was reduced in *ETV6::RUNX1*+ HPCs as indicated by lower number of expressed genes per cell (n_genes) and unique transcripts detected per cell (n_UMIs), respectively (Figure S5H,I).

Next, we performed immunophenotyping of hiPSC‐derived HPCs using an antibody panel designed to identify hematopoietic progenitors in human bone marrow^39^ (gating strategy depicted in Figure S7). *ETV6::RUNX1* expression led to increased numbers of phenotypic hematopoietic stem cells (HSC: CD34+LIN‐CD38‐CD90+CD45RA‐, Figure 3G), in keeping with expansion of naïve progenitors observed by scRNA‐seq (padj < 0.05; Table S9). Increased persistence of preleukemic *ETV6::RUNX1*+ HSCs has been reported previously.^22^, ^40^

The majority of HPCs were characterized as multipotent progenitors (MPP: LIN‐CD34+CD38‐CD90‐CD45RA‐) by flow cytometry, while scRNA‐seq analysis revealed a large proportion of megakaryocyte‐ and erythrocyte‐committed HPCs. This discordance between transcriptionally and immunophenotypically defined cell types underlines the strength of scRNA‐seq for in‐depth characterization of cellular states and detection of differentiation trajectories. Correlating protein and mRNA levels would require further analyses that combine proteome and transcriptome profiling on single‐cell level, such as cellular indexing of transcriptomes and epitopes by sequencing (CITE‐seq).

In colony forming assays, *ETV6::RUNX1*+ HPCs exclusively formed granulocyte‐macrophage progenitor colonies (CFU‐GM; Figure S8), while wild‐type HPCs were also able to differentiate into common myeloid progenitor colonies (CFU‐GEMM) and erythroid progenitors (BFU‐E). This underlines the increased granulocyte/monocyte/DC lineage commitment that we observed in *ETV6::RUNX1*+ HPCs by scRNA‐seq.

In summary, our preleukemic *ETV6::RUNX1*+ hiPSC model recapitulates the accumulation of phenotypic HSCs and exhibits increased commitment towards the granulocyte/monocyte/DC lineage during early hematopoiesis, as revealed by scRNA‐seq analysis.

### ETV6::RUNX1 induces *H1‐0* promoter activation

**Figure 4:**
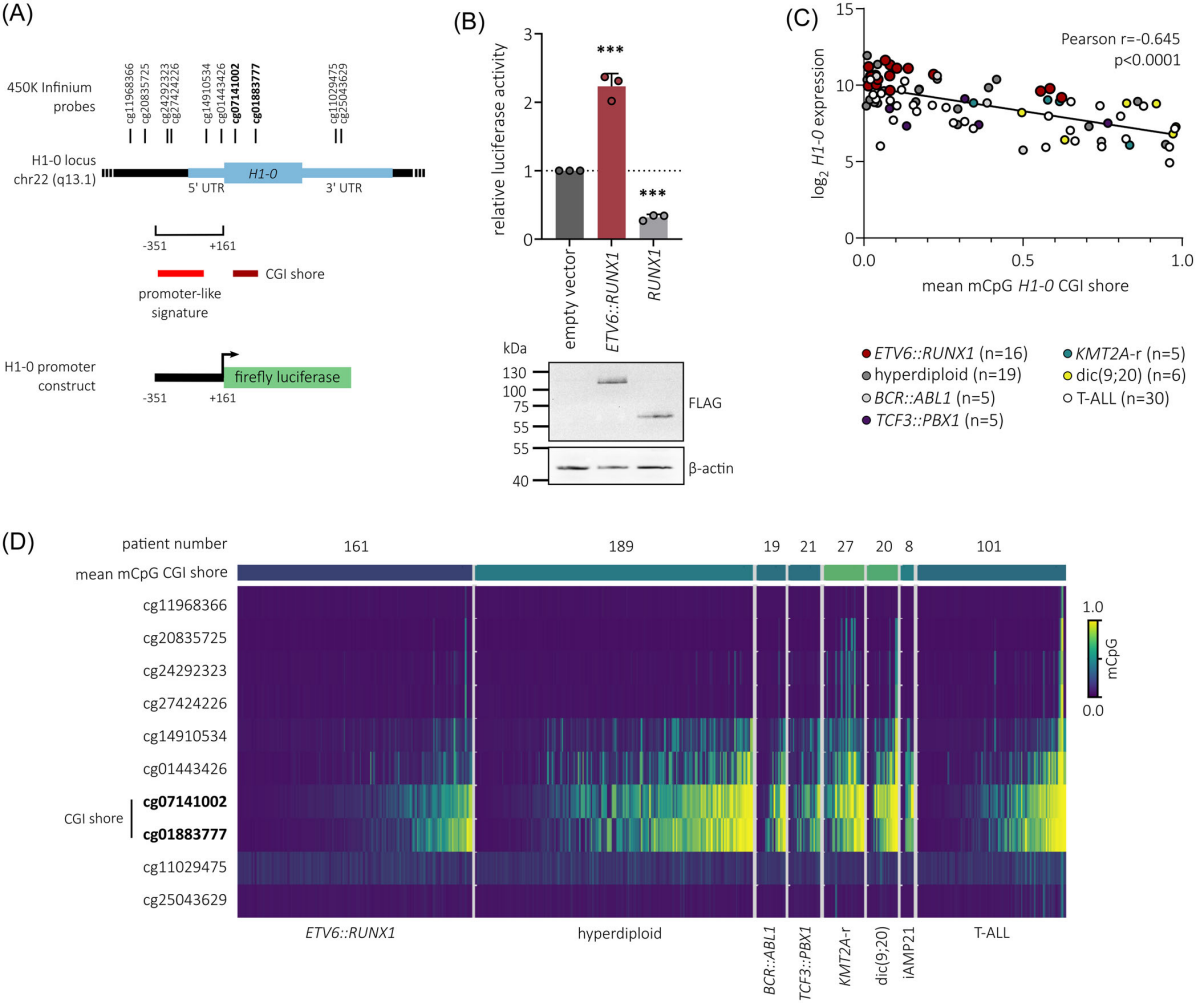
**ETV6::RUNX1 induces** ***H1‐0*** **promoter activation**. **(A)** Schematic representation of the *H1‐0* locus, including the 512‐bp region (nucleotides −351 to +161 from TSS) encompassing promoter‐like signature EH38E2163184 (ENCODE). The *H1‐0* CpG island (CGI) shore and 450K Infinium array probes are indicated. **(B)** 293T cells were transfected with a vector encoding the *H1‐0* promoter‐like signature indicated in **(A)**, together with the empty pcDNA3.1 vector or pcDNA3.1 expressing either ETV6::RUNX1 or RUNX1, and a vector expressing Renilla luciferase. Luciferase activities were normalized to Renilla luciferase activity and the empty vector control. Data represent mean values of three independent replicates ± standard deviation. Significance was calculated using an ordinary one‐way ANOVA (***p < 0.001). Representative protein levels of ETV6::RUNX1, RUNX1, and β‐actin determined by Western blot are shown. **(C)** Pearson correlation of *H1‐0* expression and mean DNA methylation of the *H1‐0* CGI shore probes cg07141002 and cg01883777 in leukemia patients (accession number GSE49032^41^). Expression is shown for microarray probe 208886_at. Each dot represents a single patient. **(D)** *H1‐0* DNA methylation in different leukemia entities is visualized as a heatmap with each column corresponding to a single patient (accession number GSE49032^41^). Within each entity, patients are sorted according to mean DNA methylation of CGI shore probes cg07141002 and cg01883777. The total number of patients per entity is indicated.

Given that our findings show strong association between ETV6::RUNX1 and H1‐0 expression, we tested the potential of ETV6::RUNX1 to transactivate the *H1‐0* promoter. To this end, we cloned the *H1‐0* promoter region (−351 to +161 from TSS) into a luciferase reporter plasmid (Figure 4A), which was transfected into 293T cells along with either an empty vector or vectors containing FLAG‐tagged *ETV6::RUNX1* or *RUNX1* sequences. Luciferase activity measurements confirmed that expression of ETV6::RUNX1 is sufficient to activate the *H1‐0* promoter (2.2‐fold), while RUNX1 expression reduced luciferase activity (3.1‐fold; Figure 4B). However, our previous analyses in murine cells^8^, ^9^ and analysis of the *H1‐0* promoter region using published chromatin immunoprecipitation sequencing (ChIP‐seq) data of *ETV6::RUNX1*+ REH cells^42^, ^43^ did not show direct binding of either the fusion protein or RUNX1 to the *H1‐0* promoter region or distal enhancer regions upstream of *H1‐0* (Figure S9A), suggesting an indirect mechanism of H1‐0 upregulation upon ETV6::RUNX1 expression.

Additionally to transcriptional control via binding of transcription factors, differential DNA methylation of the *H1‐0* CpG island (CGI) shore has been reported to regulate H1‐0 expression in various solid tumor types, acting as an enhancer element.^27^ Hence, we analyzed previously published 450K Infinium microarray DNA methylation data comprising patient samples of T‐ALL and six B‐ALL subtypes^41^ (*n* = 546). Indeed, the mean CGI shore methylation of *H1‐0*, comprising probes cg07141002 and cg01883777, inversely correlated with *H1‐0* expression (Pearson *r* = −0.645, *p* < 0.0001; Figures 4C and S9B) and was lowest in *ETV6::RUNX1*+ BCP‐ALL (Figure 4D), indicating that H1‐0 expression is regulated via dynamic methylation of its CGI shore in leukemia. While the connection of ETV6::RUNX1 and H1‐0 remains correlative, our data suggest that ETV6::RUNX1 induces upregulation of H1‐0 in an indirect manner, possibly via the *H1‐0* promoter and CGI shore region.

### H1‐0 levels decrease during hematopoiesis

**Figure 5:**
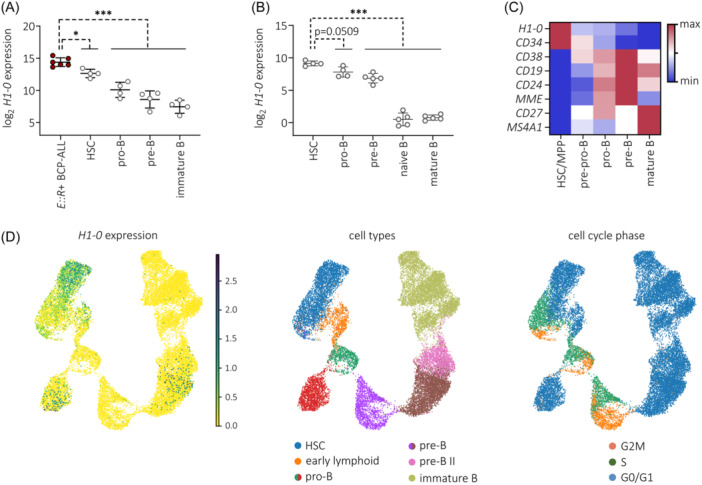
**H1‐0 expression decreases during hematopoiesis**. **(A)** *H1‐0* expression in *ETV6::RUNX1*+ BCP‐ALL (*n* = 6) and healthy B cell precursor stages derived from a published RNA‐seq dataset (accession number GSE115656^45^). B cell precursor fractions are HSCs (CD34+CD19‐IgM‐), pro‐B cells (CD34+CD19+IgM‐), pre‐B cells (CD34‐CD19+IgM‐) and immature B cells (CD34‐CD19+IgM+). **(B)** *H1‐0* expression in healthy B cell precursor stages derived from a published expression microarray dataset (accession number GSE24759^46^). B cell precursor fractions are HSCs (CD34+CD38‐), pro‐B cells (CD34+CD10+CD19+), pre‐B cells (CD34‐CD10+CD19+), naïve B cells (CD19+IgD+CD27‐), and mature B cells (CD19+IgD+CD27+). **(B, C)** Mean expression ± standard deviation is indicated and data was analyzed for statistical significance using an ordinary one‐way ANOVA (*p < 0.05, ***p < 0.001). **(C)** Min–max‐normalized mean expression per cell type derived from a fetal liver scRNA‐seq dataset (accession number E‐MTAB‐7407^47^). **(D)** *H1‐0* expression levels across normal B‐lymphoid differentiation distinguishing cell cycle status is depicted in a scRNA‐seq UMAP visualization of B cell precursor cells from bone marrow of eight healthy donors.^48^

During hematopoiesis, H1‐0 is expressed in undifferentiated, quiescent progenitor cells.^44^ To characterize *H1‐0* expression during B lymphopoiesis, we analyzed published RNA‐seq data from adult and pediatric bone marrow,^45^ expression microarray data from umbilical cord blood and peripheral blood,^46^ and scRNA‐seq data from fetal liver.^47^ Across these datasets, we observed a continuous decrease of *H1‐0* expression during B cell development (Figure 5A–C) and significant upregulation of *H1‐0* in *ETV6::RUNX1*+ ALL cells (*n* = 6) compared to HSCs and later B cell progenitor stages (Figure 5A).

To examine *H1‐0* expression in the context of cell cycle activity and hematopoietic differentiation, we employed scRNA‐seq data of B precursor cells derived from adult bone marrow.^48^ *H1‐0*+ cell numbers decreased along the B lineage trajectory, clustering preferentially to G0/G1 cell cycle states, especially within the HSC, early lymphoid and pro‐B populations (Figure 5D). Taken together, our data suggest that H1‐0 is an indicator of differentiation state.

### H1‐0 is a key mediator of the ETV6::RUNX1‐specific gene signature

**Figure 6:**
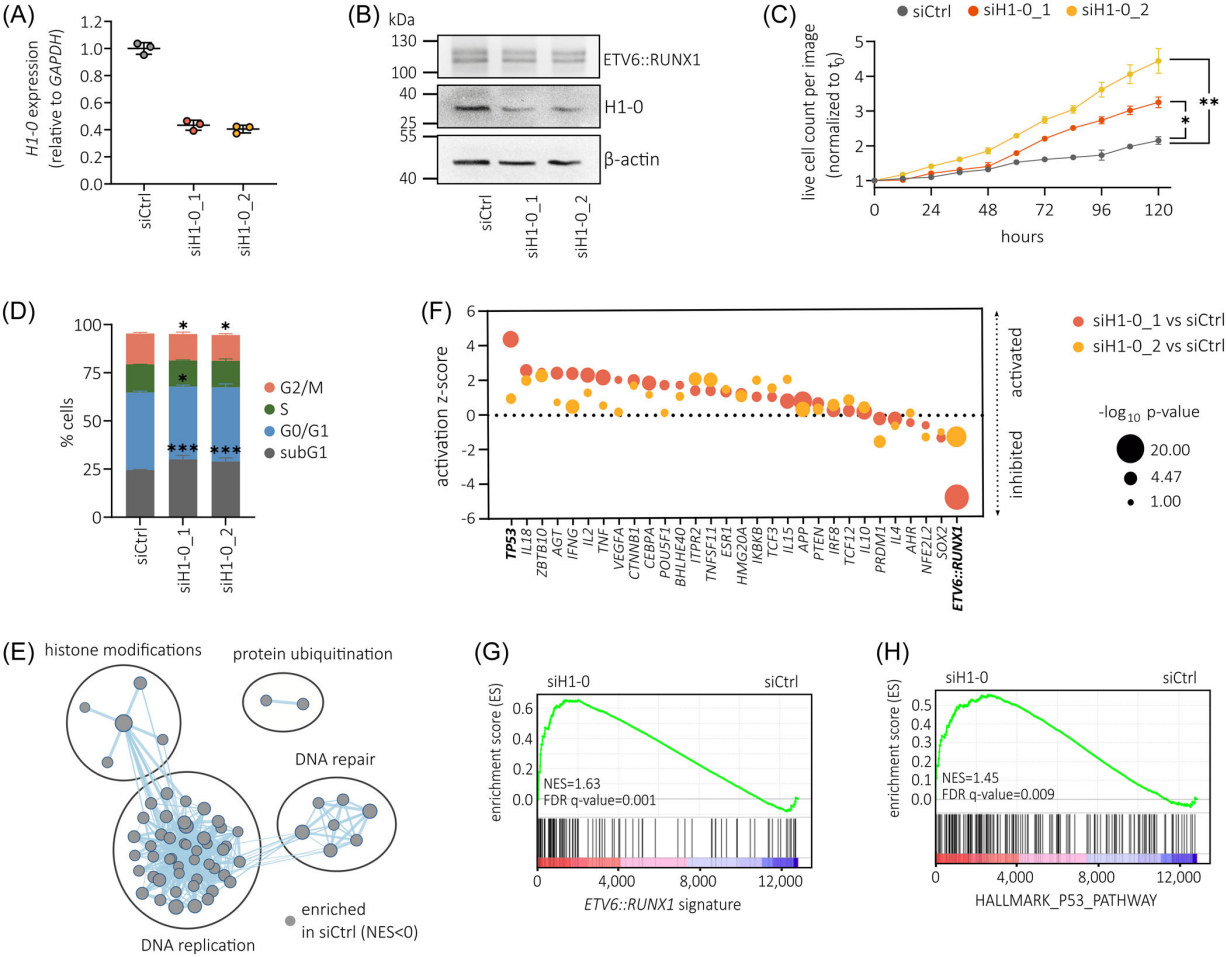
**H1‐0 is a key mediator of the ETV6::RUNX1‐specific gene signature**. *H1‐0* expression determined by **(A)** RT‐qPCR and **(B)** representative Western blot of REH cells treated for 48 hours with a non‐targeting siRNA pool (siCtrl) or *H1‐0*‐targeting siRNA pools siH1‐0_1 or siH1‐0_2. Data is presented as the mean ± standard deviation. **(C)** IncuCyte proliferation assay of REH cells treated with siCtrl or *H1‐0*‐targeting siRNAs (siH1‐0_1 and siH1‐0_2). Values represent the mean of 12 wells ± SEM, and were normalized to time point zero (cell seeding). Significance was determined by repeated measures one‐way ANOVA (*p < 0.05, **p < 0.01). **(D)** Cell cycle distribution of REH cells treated with siCtrl or *H1‐0*‐targeting siRNAs (siH1‐0_1 and siH1‐0_2) determined by Nicoletti assay. Data is presented as the mean + standard deviation. Statistical significance was analyzed by two‐way ANOVA comparing siH1‐0 to siCtrl treatment (*p < 0.05, ***p < 0.001). **(E)** Ingenuity Pathway analysis (IPA, Qiagen) of upstream regulators significantly enriched in both siH1‐0_1 versus siCtrl and siH1‐0_2 versus siCtrl (*p* < 0.05). **(F)** GSEA results of siH1‐0 versus siCtrl using a published gene set of 103 significantly upregulated genes^49^ in both REH and AT‐2 cells upon ETV6::RUNX1 knockdown (cut‐offs: log2 fold change > 0.9 and adjusted *p* < 0.05). Normalized enrichment score (NES) and FDR are indicated. **(G)** GSEA of siH1‐0 versus siCtrl using the HALLMARK_P53_PATHWAY gene set derived from Human MSigDB Collections. **(H)** Enrichment map of gene sets enriched in siCtrl REH cells compared to siRNA‐mediated knockdown of H1‐0 (cut‐offs: *p* < 0.005, FDR *q*‐value < 0.1) using the canonical pathways gene set collection (Human MSigDB Collections). No significantly enriched gene sets were found in siH1‐0 REH cells using the indicated cut‐offs. Groups of similar pathways are indicated.

To determine the contribution of H1‐0 to *ETV6::RUNX1*+ BCP‐ALL pathology, we knocked down H1‐0 in the *ETV6::RUNX1*+ BCP‐ALL cell line REH and performed gene set enrichment analysis (GSEA) on RNA‐seq data. Knockdown reduced *H1‐0* RNA expression by ≈2.4‐fold, translating to decreased protein levels compared to non‐targeting siRNA (siCtrl) treatment (Figure 6A,B). Cell proliferation increased upon H1‐0 knockdown (Figure 6C) along with a rise in apoptotic subG1 cells and reduced frequency of cells in G2/M (Figure 6D). GSEA using the canonical pathways collection (Human MSigDB Collections) revealed significant enrichment (cut‐offs: *p* < 0.005, FDR *q* value of < 0.1) of gene signatures associated with DNA replication, histone modification, DNA repair and protein ubiquitination in siCtrl‐treated REH cells (Figure 6E and Table S10), while no gene sets were identified as significantly enriched in REH cells treated with *H1‐0*‐targeting siRNA using the same cut‐offs (Table S11). Notably, GSEA detected enrichment of gene sets linked to histone acetylation (Table S10 [in red]) in siCtrl‐treated REH cells, consistent with previous reports highlighting strong correlation between H1‐0 expression and chromatin acetylation.^50^, ^51^

To ascertain common molecular drivers of gene expression changes observed upon H1‐0 knockdown, we applied upstream regulator analysis using the Ingenuity Pathway Analysis (IPA) suite^52^ (Qiagen). Interestingly, the most significant potential driver detected by IPA upstream regulator analysis was ETV6::RUNX1, with *p* = 3.7 × 10^−16^ (for siH1‐0_1 vs. siCtrl, Table S12) and *p* = 3.5 × 10^−11^ (for siH1‐0_2 vs. siCtrl, Table S13) respectively (Figure 6F). Negative activation z‐scores indicated inhibition of the ETV6::RUNX1 transcription factor upon H1‐0 knockdown. Given that ETV6::RUNX1 primarily functions as a repressor of RUNX1‐regulated genes,^53^ we employed a set of genes downregulated by ETV6::RUNX1 (cut‐offs: log2 fold change > 0.9 and *p* < 0.05)^49^ to validate our findings. Indeed, GSEA revealed significant upregulation of these ETV6::RUNX1 signature genes upon H1‐0 knockdown (normalized enrichment score [NES] = 1.63, FDR *q* value = 0.001; Figure 6G and Figure S10A).

Furthermore, we detected significant activation of *TP53* (encoding for the tumor suppressor p53) following H1‐0 knockdown, as indicated by both upstream regulator analysis (Figure 6F) and GSEA (NES = 1.45, FDR *q* value = 0.009; Figure 6H). Indeed, previous studies have demonstrated that ETV6::RUNX1 suppresses p53 activity by upregulating MDM2.^54^ Accordingly, we detected downregulated *MDM2* in REH cells upon H1‐0 knockdown (Figure S10B). Moreover, both *EPOR* and *RAG1*, two genes upregulated by ETV6::RUNX1 and key factors in *ETV6::RUNX1*+ BCP‐ALL pathophysiology,^6^, ^42^, ^55^, ^56^ exhibited reduced levels upon H1‐0 knockdown (Figure S10B) as well as significant correlation with *H1‐0* RNA expression in *ETV6::RUNX1*+ BCP‐ALL patient samples derived from the PeCan St. Jude cohort^30^, ^31^ (n = 87, Figure S10C). Taken together, these data imply that linker histone H1‐0 is a novel key regulator of ETV6::RUNX1‐induced expression changes.

### H1‐0 inducer Quisinostat synergizes with frontline drugs in *ETV6::RUNX1*+ leukemic cells

**Figure 7:**
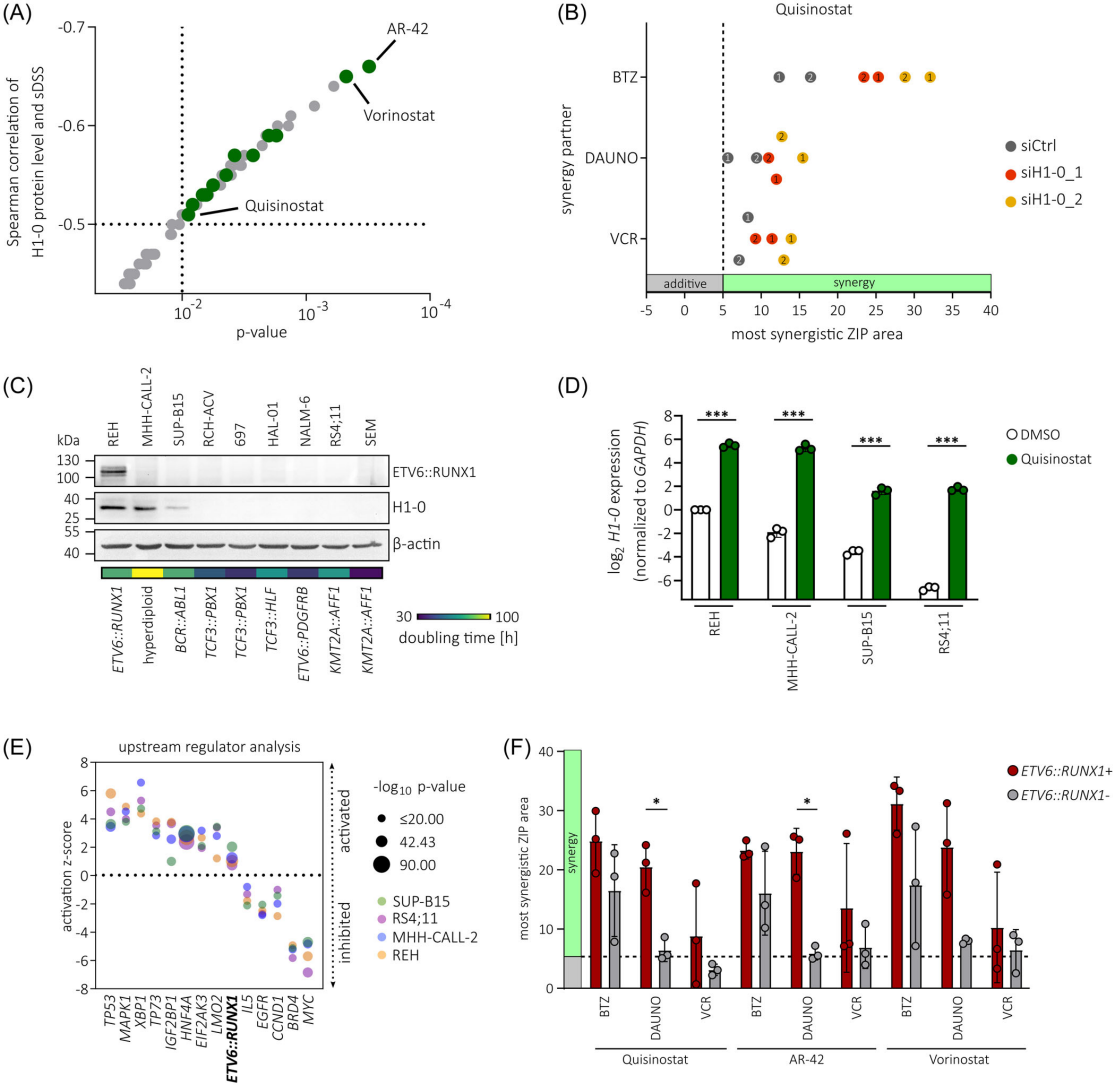
**H1‐0 inducer Quisinostat synergizes with frontline drugs in** ***ETV6::RUNX1*** + **BCP‐ALL**. **(A)** Spearman correlation and Spearman p‐values of H1‐0 protein levels and selective drug sensitivity scores (sDSS) in 25 BCP‐ALL cell lines derived from the FORALL platform^57^, ^58^ (https://proteomics.se/forall/; cut‐offs: *p* < 0.05, FDR < 0.25). HDACis are marked in green. **(B)** Most synergistic area scores (2 × 2 dose window) of Quisinostat with Vincristine (VCR), Daunorubicin (DAUNO) or Bortezomib (BTZ) are indicated. REH cells were treated with drug combinations 48 h after siRNA treatment. Biological replicates are indicated. **(C)** Protein levels of ETV6::RUNX1, H1‐0 and β‐actin in BCP‐ALL cell lines were quantified by Western blot. Chromosomal aberrations present in the respective cell line are indicated. Doubling times are derived from the DSMZ (https://www.dsmz.de/). **(D)** RT‐qPCR quantifying *H1‐0* levels 24 h after treatment with DMSO or 1 µM Quisinostat in BCP‐ALL cell lines. Values represent mean ± standard deviation from three independent replicates and data was analyzed for statistical significance using an ordinary one‐way ANOVA (***p < 0.001). **(E)** Activation z‐scores of upstream regulator signatures overlapping between BCP‐ALL cell lines treated with 1 µM Quisinostat versus DMSO for 24 h. **(F)** Bar graph of mean most synergistic ZIP area scores of *ETV6::RUNX1*+ and *ETV6::RUNX1*‐ BCP‐ALL cell lines and PDX samples. Mean ± standard deviation is indicated. Statistical significance was determined by t test with Welch's correction (*p < 0.05).

Due to their cytostatic activity, HDACis are potent inducers of H1‐0 expression.^50^, ^51^ Importantly, H1‐0 has been identified as a mediator of the antitumor effect induced by the pan‐HDACi Quisinostat in various solid cancers.^51^ Hence, basal expression levels of H1‐0 could be a marker for HDACi activity in BCP‐ALL. Indeed, we found a striking inverse correlation between H1‐0 protein levels and sensitivity towards HDACis in a panel of 25 BCP‐ALL cell lines (data derived from the Functional Omics Resource of Acute Lymphoblastic Leukemia [FORALL] platform, https://proteomics.se/forall/ ^57^, ^58^), in particular with AR‐42 and Vorinostat (*p* < 0.001), as well as 11 other HDACis, including Quisinostat (*p* < 0.01; Figure 7A). To examine the effect of H1‐0 knockdown on Quisinostat sensitivity in *ETV6::RUNX1*+ BCP‐ALL, REH cells were transduced with *H1‐0*‐targeting siRNA for 48 h and treated with Quisinostat (Figure S11A,B). While downregulation of H1‐0 did not alter sensitivity towards single drug treatment with Quisinostat (Figure S11C), combination with the commonly used B‐ALL chemotherapeutics Vincristine and Daunorubicin, or the proteasome inhibitor Bortezomib, increased drug synergism (Figure 7B).

To confirm the H1‐0‐inducing properties of Quisinostat, we again performed *H1‐0* promoter luciferase assays in 293 T cells and treated with increasing sub‐lethal concentrations of Quisinostat for 24 h. Indeed, Quisinostat induced *H1‐0* promoter activation (Figure S11D) and an increase of endogenous *H1‐0* levels (Figure S11E) in a dose‐dependent manner. Moreover, BCP‐ALL cell lines with varying levels of basal H1‐0 expression (high levels: REH and MHH‐CALL‐2, medium level: SUP‐B15, low level: RS4;11) strongly upregulated *H1‐0* upon treatment with 1 µM Quisinostat for 24 h (Figure 7C,D). As anticipated, basal H1‐0 levels reflected doubling times of BCP‐ALL cell lines (Figure 7C) and REH cells were most resistant to Quisinostat treatment, as indicated by lowest fraction of subG1 cells after 24‐hour Quisinostat treatment (Figure S11F). Transcriptome analysis revealed a drug‐induced shift in all four BCP‐ALL cell lines and dysregulation of similar signaling pathways (Figure S11G–I and Tables S14–S17). These include inhibition of *MYC* ^59^, ^60^ and *BRD4*,^61^ as described previously, as well as the activation of *TP53* signaling due to induction of apoptosis (Figure 7E). Interestingly, the *ETV6::RUNX1* signature was activated upon Quisinostat treatment, indicating a connection of histone acetylation and ETV6::RUNX1 target gene transcription, that has also been proposed previously.^20^

We further performed synergy drug screens with Quisinostat, as well as AR‐42 and Vorinostat, the two HDACis that showed highest anti‐correlation with H1‐0 protein levels (Figure 7A). For this, we screened three *ETV6::RUNX1*‐ BCP‐ALL cell lines (MHH‐CALL‐2, SUP‐B15, and RS4;11) and the *ETV6::RUNX1*+ cell line REH as well as two *ETV6::RUNX1*+ PDX samples (Figures 7F and S12). Overall, simultaneous inhibition of HDACs and the proteasome showed high synergy. Clinical efficacy of combination treatment with Bortezomib and HDACis has been shown in previous studies targeting hematologic malignancies.^62^ Moreover, we found high synergy in *ETV6::RUNX1*+ samples using HDACis in combination with the topoisomerase II inhibitor Daunorubicin, while there was no or low synergy in *ETV6::RUNX1*− samples using this combination. Of note, Quisinostat induced effective killing in BCP‐ALL cell lines at much lower concentration range (0.2–10 nM) than AR‐42 (10–1000 nM) or Vorinostat (100–5000 nM; Tables S18S–20). Taken together, these analyses indicate that combinatorial drug treatment using Quisinostat in combination with Daunorubicin or Bortezomib might be beneficial for targeting *ETV6::RUNX1*+ leukemic cells.

## DISCUSSION

In this study, we established preleukemic *ETV6::RUNX1*+ knock‐in hiPSC models derived from two donors. Transcriptome analysis of these models revealed that ETV6::RUNX1 expression upregulates H1‐0, a variant of the H1 linker histone family that promotes chromatin compaction.^26^, ^63^ We demonstrate that H1‐0 regulates cellular quiescence and significantly contributes to the repressive expression signature conferred by ETV6::RUNX1. Moreover, we found that H1‐0 downregulation increased drug synergism of the HDACi Quisinostat with common B‐ALL chemotherapeutics.

High H1‐0 levels observed in HSCs are in line with the largely quiescent nature of these cells.^44^ The progressive decrease of H1‐0 levels during hematopoiesis supports the notion that H1‐0 accumulates in quiescent cells that have high proliferative capacity.^27^, ^34^ Increased quiescence of *ETV6::RUNX1*+ preleukemic cells is in keeping with our previous detection of these cells in cord blood of approximately 5% of healthy newborns,^2^ offering a potential explanation for prolonged latency periods of *ETV6::RUNX1*+ leukemia, which can extend up to 14 years.^64^

The relationship between ETV6::RUNX1 and H1‐0 remains correlative, and further studies are needed to explore the role of chromatin compaction and histone acetylation in BCP‐ALL development, as well as the impact of H1‐0 during hematopoietic differentiation, to establish a clearer connection. Interestingly, increased H1‐0 levels were also observed in leukemic BCP‐ALL patient samples, suggesting retention of chromatin compaction throughout *ETV6::RUNX1*+ BCP‐ALL development. This is consistent with a recent report of reduced global chromatin accessibility in *ETV6::RUNX1*+ BCP‐ALL compared to other ALL subtypes.^65^ Similar loss of chromatin accessibility and cell cycle arrest has been detected in myeloid progenitors harboring the *RUNX1::ETO* translocation that retains the DNA‐binding RHD, allowing it to bind to RUNX1 target sites.^66^

Aberrant co‐expression of myeloid genes has been previously identified in preleukemic *ETV6::RUNX1*+ pro‐B cells^10^ and we show here that early hematopoiesis of *ETV6::RUNX1*+ hiPSCs is skewed towards myeloid lineage precursors, specifically towards granulocyte/monocyte/DC commitment. It is conceivable that this myeloid bias induced by ETV6::RUNX1 impedes B lineage commitment, highlighting the need for second hit mutations for the expansion of B lineage cells. In future studies, our model could be used to study the effect of common secondary mutations (such as deletions of *PAX5*, *CDKN2A*, or the second *ETV6* allele) on BCP‐ALL development.

The H1‐0 inducer Quisinostat has demonstrated high potency and bioavailability at low nanomolar concentrations,^67^, ^68^ while preserving normal stem cell function.^51^, ^69^, ^70^ However, the predominantly cytostatic activity of HDACis in vivo suggests that single‐drug treatment is not sufficient to induce cancer remission. Using leukemic cell lines and PDX models, we show here that combinatorial treatment using the pan‐HDACi Quisinostat is a promising approach to enhance treatment of *ETV6::RUNX1*+ BCP‐ALL when administered alongside Daunorubicin or Bortezomib. Indeed, combination of Bortezomib with Quisinostat showed favorable treatment outcomes in a multiple myeloma mouse model,^71^ and a previous study also reported efficacy of other pan‐HDACis used in combination with Bortezomib in preclinical B‐ALL models, particularly in relapsed ALL.^72^ While the majority of *ETV6::RUNX1*+ BCP‐ALL patients responds well to current treatment protocols, relapse still occurs in approximately 5% of patients.^7^ Upon relapse, combination therapy with Quisinostat may serve as an alternative treatment option, especially in patients who fail to respond to bispecific antibodies such as Blinatumomab (CD19/CD3).

In conclusion, our data demonstrate that H1‐0 contributes to quiescence of *ETV6::RUNX1*+ cells. Unraveling mechanisms involved in quiescence of *ETV6::RUNX1*+ preleukemic cells may offer new opportunities for enhancing patient treatment.

## AUTHOR CONTRIBUTIONS

**Vera H. Jepsen**: Conceptualization; investigation; data curation; validation; methodology; formal analysis; visualization; writing—original draft; writing—review and editing. **Andrea Hanel**: Formal analysis; software; methodology; writing—review and editing. **Daniel Picard:** Formal analysis; software. **Rigveda Bhave**: Investigation; methodology; resources. **Rebecca Hasselmann**: Investigation; formal analysis; visualization; writing—review and editing. **Juha Mehtonen**: Formal analysis. **Julian Schliehe‐Diecks**: Investigation; formal analysis; methodology. **Carla‐Johanna Kath:** Investigation; methodology. **Vithusan Suppiyar:** Formal analysis; software. **Yash Prasad:** Formal analysis; software. **Katerina Schaal**: Investigation. **Jia‐Wey Tu**: Investigation. **Nadine Rüchel:** Resources. **Ersen Kameri**: Resources. **Nan Qin**: Resources. **Herui Wang**: Resources. **Zhengping Zhuang**: Resources. **Rabea Wagener**: Data curation; formal analysis. **Lena Blümel**: Resources. **Tobias Lautwein**: Formal analysis; software. **Daniel Hein**: Supervision; funding acquisition; conceptualization. **David Koppstein:** Supervision; resources. **Gesine Kögler**: Resources. **Marc Remke**: Resources. **Sanil Bhatia**: Supervision; resources; writing—review and editing. **Merja Heinäniemi**: Supervision; writing—review and editing. **Arndt Borkhardt**: Funding acquisition; supervision; writing—review and editing. **Ute Fischer**: Funding acquisition; conceptualization; supervision; writing—review and editing.

## CONFLICT OF INTEREST STATEMENT

The authors declare no conflict of interest.

## FUNDING

This work was funded by the German Research Foundation (DFG, 495318549, GRK2578: 417677437), German Cancer Aid (Deutsche Krebshilfe, 70114736), Deutsche José‐Carreras Leukämie‐Stiftung (DJCLS, 18R/2021), Deutsche Kinderkrebsstiftung (DKKS, A2023/31), the German Ministry for Education and Research (BMBF, 01KD2410A (EDI‐4‐ALL)), the German Federal Office for Radiation Protection (BfS, 3622S32231), the Parents' initiative Löwenstern e.V., and the Katharina Hardt‐Stiftung.

## Supporting information

Supporting information.

Supporting information.

## Data Availability

The datasets produced in this study are available in the following databases:
RNA‐seq data: Gene Expression Omnibus GSE270944 and GSE283119. scRNA‐seq data: Gene Expression Omnibus GSE270945.
