# Supplementary material for: H1‐0 is a specific mediator of the repressive ETV6::RUNX1 transcriptional landscape in preleukemia and B cell acute lymphoblastic leukemia
Source: Hemasphere. 2025 Apr 2;9(4):e70116. doi: 10.1002/hem3.70116 (PMC11962653; doi:10.1002/hem3.70116)
Supplement: Supplementary file 1 — Supporting information. [file HEM3-9-e70116-s001.docx]

**H1-0 is a specific mediator of the repressive ETV6::RUNX1 transcriptional landscape in preleukemia and B cell acute lymphoblastic leukemia**

**Supplementary Methods**

**Patient-derived xenografts**

Patient blasts were injected intravenously into 6-week-old NSG mice (The Jackson Laboratory) and engraftment was assessed regularly by flow cytometric detection of human CD45+ cell percentage (BioLegend, #304011) in peripheral blood starting four weeks post injection^1^. Mice were sacrificed at predetermined timepoints and human CD45+ cells were isolated from bone marrow and spleen using the mouse cell depletion kit (Miltenyi Biotec, #130-104-694) to achieve >90% purity of human cells.

**Cell culture**

hiPSCs were cultured at 37 °C and 5% CO_2_ on Geltrex-coated (Thermo Fisher Scientific, #A1413301) culture plates in mTeSR Plus (Stemcell Technologies, #100-0276) and were passaged in aggregates every 3-5 days using Versene solution (Thermo Fisher Scientific, #15040066) according to the manufacturer’s instructions. hiPSC lines were cultured for a maximum of 40 passages. Colony morphology was examined by microscopy (Axiovert 200, Zeiss). Pluripotency marker expression was assessed every 10 passages by flow cytometry (PE anti-human SSEA-4 antibody, BioLegend, #330405) or RT-qPCR for *DNMT3B*, *GDF3*, *POU5F1* and *NANOG*. Chromosomal integrity of hiPSCs was confirmed by karyotyping at the Institute of Human Genetics (Hannover Medical School (MHH), Germany).

BCP-ALL cell lines were maintained at 37 °C and 5% CO_2_ in RPMI GlutaMAX (Thermo Fisher Scientific, #61870036) with 20% fetal bovine serum (Biowest, #S181H-500). 293T cells were cultured at 37 °C and 5% CO_2_ in DMEM GlutaMAX (Thermo Fisher Scientific, #31966) with 10% fetal bovine serum. All cell lines were routinely tested for mycoplasma using the Venor GeM Advance mycoplasma detection kit (Minerva Biolabs, #11‑7024). Short tandem repeat-based authentication of cell lines was performed at the Genomics and Transcriptomics Laboratory (GTL, Heinrich Heine University Düsseldorf, Germany) using the GenePrint 10 system (Promega, #B9510).

**Genotyping PCRs**

Genomic DNA was isolated from puromycin-resistant hiPSC colonies using the QIAamp DNA Blood Mini kit (Qiagen, #51104). To ensure correct incorporation of the *RUNX1* HDR template into the *ETV6* locus, genotyping PCRs were performed with two sets of primers (5’-fw and 5’‑rev, 3’-fw and 3’-rev). To confirm heterozygous allele status of *ETV6::RUNX1*, a third PCR combining primers 5’-fw, 3’-rev and 5’-fw2 was performed. Primer sequences are listed in Table S21. Genotyping results were confirmed by Sanger sequencing, performed at the GTL (Heinrich Heine University Düsseldorf, Germany).

**Quantitative real-time PCR (RT-qPCR)**

RNA was extracted with the RNeasy Mini kit (Qiagen, #74104) according to the manufacturer’s instructions. On column DNA digestion was performed using the RNase free DNase Set (Qiagen, #79254). For RT-qPCR detection of *H1-0*, an additional DNase digest was performed with the TURBO DNA-free kit (Thermo Fisher Scientific, #AM1907). cDNA was synthesized from 1 µg of total RNA using M-MLV reverse transcriptase (Promega, #M1701) according to the manufacturer’s instructions. Due to limited amount of RNA following siRNA mediated *H1‑0* knockdown in REH cells, 200 ng of total RNA were reverse transcribed. RT-qPCR was performed in triplicates using SYBR Green PCR Mix (Thermo Fisher Scientific, #4309155) or TaqMan Universal PCR Master Mix (Thermo Fisher Scientific, #4304437) and fluorescence was measured with the CFX384 Touch Real-Time PCR Detection System (Bio-Rad). *GAPDH*, *ATP5PB* and *PGK1* served as housekeeping genes for normalization and have previously been confirmed as top-ranking housekeeping genes during iPSC reprogramming^2^. A list of RT‑qPCR primers used in this study can be found in Table S21. Relative quantification of expression was calculated using the ∆∆Ct method. RT-qPCR primer efficiencies were validated by performing dilution series (85-110% efficiency, R2>0.98) and melt curve analyses^3^. No‑template control (NTC) and no-reverse-transcriptase controls (NRT) were included on each plate.

**Flow cytometry**

For immunophenotyping of hematopoietic progenitor cells (HPCs) differentiated from hiPSCs, the following combinations of cell surface markers were used (as previously described by Pellin *et al*.^4^). Hematopoietic stem cells (HSC): LIN-CD34+CD38-CD90+CD45RA-; multipotent progenitors (MPP): LIN-CD34+CD38-CD90-CD45RA-; multi-lymphoid progenitors (MLP): LIN-CD34+CD38-CD90-CD45RA+. The gating strategy is summarized in Figure S6.

Stained HPCs were measured with a CytoFLEX S cytometer (Beckmann Coulter). Lineage positive cells were identified by staining with a lineage cocktail with addition of CD15. The following antibodies were used: LIN BV510 (#348807), CD15 BV510 (#323028), CD90 FITC (#328108), CD135 PE (#313306), CD10 PE/Cy7 (#312214), CD7 APC (#982702), CD45RA APC/Fire750 (#304152), CD34 PB (#343512), all from BioLegend. CD38 BV650 (#569966) from BD Biosciences. Dead cells were excluded by staining with 7-AAD viability dye (#A07704) from Beckmann Coulter.

Cell cycle profiles were generated by performing Nicoletti assays^5^. Briefly, DNA was stained with propidium iodide after partial cell lysis in hypotonic buffer (0.1% sodium citrate, 0.1% Triton X-100, 0.5 mg/ml RNase A containing 40 µg/ml propidium iodide) and stained cells were measured with a CytoFLEX S cytometer (Beckmann Coulter).

Flow cytometry data were analyzed with FlowJo v10.8. Uniform Manifold Approximation and Projection (UMAP) visualizations were generated using the UMAP plugin for FlowJo.

**Colony-forming unit (CFU) assay**

To determine differentiation potential of hiPSC-derived HPCs, 1000 HPCs were seeded per well of a 6-well plate in MethoCult SF H4636 methyl-cellulose medium (#04636, Stemcell Technologies) and cultivated at 37°C and 5% CO_2_ for 14 days. The resulting colonies were counted under microscopic guidance (Keyence BZX-800), distinguishing red (burst/colony-forming unit erythrocytes [B/CFU-E]), white (CFU granulocyte/macrophages [CFU-GM]) or mixed colonies (CFU granulocytes/erythrocytes/ macrophages/monocytes [CFU-GEMM]).

**IncuCyte proliferation assay**

To assess cell proliferation upon *H1-0* downregulation, 5000 REH cells/well were seeded onto poly-L-ornithine-coated (0.01% solution, #A4957, Sigma-Aldrich) 384-well plates (Corning, #781091) following siRNA treatment. Pictures were taken every 12 hours using an IncuCyte S3 live cell imager (Essen Biosciences, Sartorius). Dead cells were excluded by staining with IncuCyte Cytotox Green Reagent (Essen Biosciences, Sartorius, #4633) to a final concentration of 125 nM. Live cells (low green) count per image normalized to the first timepoint was determined using the IncuCyte software (v2021C, Essen Biosciences, Sartorius) over 5 days in culture.

**Bulk RNA sequencing and data analysis**

HW8 and ChiPSC12 hiPSCs of similar passage number (±2 passages) were lysed on ice using RLT buffer (Qiagen, #79216) and detached by scraping. RNA was isolated using the RNeasy Mini kit (Qiagen, #74104) with on-column DNA digestion (Qiagen, #79254). REH cells were transfected with siRNA as described above and RNA was extracted after 48 hours. BCP-ALL cell lines were treated for 24 hours with 1 µM Quisinostat or DMSO (1:10000) and RNA was extracted as described previously. RNA quality was assessed with the 2100 Bioanalyzer system (Agilent, #G2939BA).

Sequencing of hiPSCs and siRNA-treated REH cells (accession number GSE270944) was performed at the next-generation sequencing core facility of the German Cancer Research Center (DKFZ). Barcoded libraries were prepared from 0.5 µg of total RNA using the TruSeq RNA Sample Preparation v2 kit (low-throughput protocol, Illumina) and quantified with the Bioanalyzer system (Agilent). 7.5 pM denatured libraries were used as input for cBot (Illumina) and subjected to deep sequencing using the NovaSeq 6000 (Illumina) for 101 cycles, with an additional 7 cycles for index reading. Analysis of fastq files was performed by using the Partek Flow software (Partek Incorporated, St. Louis, MO, USA). After assessing the read quality, a trimming step was performed (both ends: 13 bases at the 5’ end and 1 base at the 3’ end). One technical replicate of siH1-0_1 was excluded from the analysis since it did not pass quality control. After trimming, reads were aligned to the hg38 genome using the STAR v2.4.1d aligner. Unaligned reads were further processed using Bowtie 2 v2.2.5 aligner. Aligned reads were combined and expression was quantified against the Ensembl database (release 84) by the Partek Expectation-Maximization algorithm^6^. Partek flow default settings were used in all analyses.

Sequencing of Quisinostat-treated BCP-ALL cell lines (accession number GSE283119) was performed by Biomarker Technologies (BMK) GmbH. RNA concentration was detected with Nanodrop2000 (Thermo Fisher Scientific) and RNA integrity was confirmed with Agient2100 (Perkin Elmer LabChip GX). VAHTS Universal V8 RNA-seq Library Prep Kit for Illumina NR605 was used to construct the mRNA library in strict accordance with the protocol provided by Vazyme. Analysis of fastq files was performed by using the nf-core/rnaseq pipeline^7^ (https://nf-co.re/rnaseq/3.15.1/).

Unsupervised hierarchical clustering and heatmap visualization of samples was performed after normalizing mean expression to 0 with a standard deviation of 1 and using Pearson’s dissimilarity algorithm and average linkage in Partek Genomics Suite (Partek Incorporated). Upstream regulator analysis was performed with Ingenuity Pathway Analysis (IPA) suite^8^ using a significance cut‑off of p<0.05 (for siRNA-treated REH cells) or p<0.01 (for Quisinostat-treated BCP-ALL cell lines). Visualization of pathway networks was performed using the Cytoscape EnrichmentMap and AutoAnnotate applications^9,10^.

Gene set enrichment analysis (GSEA) was performed on processed RNA-seq data of REH cells treated with non-targeting or *H1-0*-targeting siRNA pools using the GSEA v4.2.3 software (http://software.broadinstitute.org/gsea). Genes were ranked by the GSEA software using the signal-to-noise metric. The permutation type was set to gene_set and number of permutations to 5000. Canonical pathways or hallmark gene sets were obtained from the Molecular Signatures Database (MSigDB, https://www.gsea-msigdb.org). Genes significantly upregulated upon *ETV6::RUNX1* knockdown in REH and AT-2 cells (cut-offs: log2 fold change >0.9 and adjusted p<0.05, n=103 genes) were derived from a published dataset^11^.

**Single-cell RNA sequencing and data analysis**

Following *in vitro* differentiation of hiPSCs, hematopoietic progenitor cells of 5 wells were pooled, filtered and resuspended in PBS with 0.04% BSA (Biowest). Cell viability was determined to be >70% using the BD Rhapsody Single-Cell Analysis system (BD Biosciences). Single-cell suspensions were used for single-cell droplet library generation on the 10X Chromium Controller system using the Chromium Single Cell 3’ NextGEM Reagent kit v3.1 (10X Genomics) according to the manufacturer’s instructions. Sequencing was performed on a NextSeq 2000 system (Illumina). All scRNA-seq reactions were performed at the GTL (Heinrich Heine University Düsseldorf).

Raw sequencing data was processed using the 10X Genomics CellRanger software (v6.0.2). Raw BCL-files were demultiplexed and processed to fastq files using the CellRanger mkfastq pipeline. Alignment of reads and UMI counting was performed *via* the CellRanger count pipeline to generate a gene-barcode matrix (genome version: GRCh38, Ensemble release 98). The CellRanger aggr pipeline was used for aggregation and sequencing depth normalization.

A total number of 46629 cells were obtained from scRNA-seq. Low-quality cells were excluded using a distance of 3 median absolute deviations from the median, considering transcript counts (n_UMIs; lower threshold: 6297.33 counts, upper threshold: 56676 counts) and number of genes (n_genes; lower threshold: 1510 genes). Moreover, cells with a fraction of mitochondrial RNAs exceeding 10% were filtered out^12^. Additionally, all genes that were not detected in at least 1 cell were excluded. The total number of cells obtained after filtering was 32358 (HW8 WT: 6493 cells, HW8 E::R 1: 8827 cells, ChiPSC12 WT: 6579 cells, ChiPSC12 E::R: 10459 cells).

Filtered cells were normalized for differences in sequencing depth *via* the normalize_total (target_sum = 1e4) function in Scanpy (version 1.10.1) followed by variance stabilizing log+1 transformation by applying the log1p function. Feature selection was performed on normalized counts using the Laplacian Score method from Delve benchmark (https://github.com/jranek/delve_benchmark)^13^. Subsequently, the top 4000 genes were used to compute principal components (PCs) *via* the pca function in Scanpy. PCs were corrected for cell line (i.e., HW8 or ChiPSC12) using Harmony (version 1.2.1) with theta=2. The top 50 harmony-corrected PCs were then used to calculate the neighborhood graph *via* the neighbors function (n_neighbors=30), and clustering of the graph performed using the leiden function (resolution 0.25). Uniform Manifold Approximation and Projection (UMAP) was computed using the umap function, the Force-directed graph (FDG) was calculated using the draw_graph function, and diffusion map representation of the data was generated using the diffmap function (n_comps=15) in Scanpy. For visualization purposes where indicated, gene expression values were scaled using scale (max_value=10) function in Scanpy.

Cell cycle phase was inferred by scoring the cell cycle gene set as defined by Tirosh *et al*.^14^ (from https://github.com/scverse/scanpy_usage/tree/master/180209_cell_cycle) by applying the score_genes_cell_cycle function in Scanpy. Statistical testing for differences in cell cycle phase (G1 vs S/G2M) between knock-in (KI, *ETV6::RUNX1*+) and WT cells was performed using t test from the tool propeller available from speckle (version 1.0.0)^15^ with asin normalization as recommended^16^.

For cell type annotation using cell types defined by Jardine *et al*.^17^, label transfer was performed following Seurat’s (version 5.1.0) integration-mapping pipeline^18^. The CD34+ human fetal bone marrow, fetal liver and cord blood CITE-seq dataset was downloaded from https://developmental.cellatlas.io/fetal-bone-marrow and supplemented with raw gene counts from Gene Expression Omnibus (GEO, accession number GSE166895). For cell type annotation using healthy adult bone marrow from Zhang *et al*.^19^ as reference, we used cellHarmony^20^ run *via* Altanalyze (version 2.1.4) with the reference “scTriangulate-titrated-cellHarmony-centroid.txt” from Zhang *et al*.^19^ and log-normalized (CPTT) counts per sample as input. The reference and h5ad files were downloaded from synapse (syn52600803).

For tissue-level comparison between the four iPSC samples from this study and CD34+ cells from human fetal liver, fetal bone marrow and cord blood from Jardine *et al*.^17^, the datasets were merged and normalized to target sum=1e4, followed by log1p transform in Scanpy. For dimensionality reduction, 4000 highly variable genes were selected based on raw counts using the highly_variable_genes function (flavor = seurat_v3, batch_key=study) in Scanpy. PCs were computed with the pca function, and then corrected for differences between the studies using Harmony (version 1.2.1) with theta=5. The top 50 corrected PCs were then used to compute the neighborhood graph with the neighbors function (n_neighbors=30), and UMAP representation calculated using the umap function in Scanpy.

For pseudobulk-level analysis, read counts with the same sample-cluster combination were summed together using Decoupler (version 1.7.0), requiring at least 10 cells and 50,000 reads per sample. Cluster-specific marker genes were then detected with the EdgeR (version 4.0.16)^21^ quasi-likelihood pipeline, adjusting for cell line in the linear model. Statistical testing for differential expression between KI (*ETV6::RUNX1*+) and WT samples relative to fold change of 2 was conducted using EdgeR’s glmTreat test, accounting for cell line effects in the linear model. Genes with FDR<10% were considered as significant. For visualization purposes, Log2 CPM values were calculated using the cpm (log=TRUE) function in EdgeR and z-scored were indicated. ComplexHeatmap (version 2.18.0) package was used to generate Heatmaps and Upset plot.

Code used to analyze scRNA-seq data is available at: https://github.com/andreahanel/2024_ETV6_RUNX1_iPSC_early_hematopoiesis

**Western blot**

Cell lysis was performed with RIPA lysis buffer (Thermo Fisher Scientific, #89900) supplemented with protease inhibitors (Roche, #11836145001). Protein concentration was determined using a bicinchoninic acid assay (BCA, Thermo Fisher Scientific, #23225), samples were mixed with 6X Laemmli buffer (Thermo Fisher Scientific, #J61337) and denatured at 95 °C for 5 min. 5-20 µg protein lysate were separated by SDS polyacrylamide gel electrophoresis (SDS-PAGE) using 8‑10% gels and transferred to Amersham Protran 0.45 µm nitrocellulose membranes (Merck, #GE10600012) by wet blotting using the Mini-Protean Vertical Electrophoresis system (Bio-Rad). Membranes were blocked for 1 hour with 5% BSA (Merck, #A3294) in T-BST at room temperature and incubated with primary antibodies (ETV6::RUNX1: rabbit IgG monoclonal, 1:2000, Abcam, #ab92336; ETV6: mouse monoclonal IgG, 1:2000, Santa Cruz Biotechnology, #sc-166835; H1-0: rabbit monoclonal IgG, 1:2000, Thermo Fisher Scientific, #MA5-35484 (ARC1059); ACTB: mouse monoclonal IgG2a, 1:5000, Merck, #A5316; FLAG: mouse IgG1 monoclonal, M2, 1:2000, Sigma-Aldrich, #F1804) diluted in blocking buffer overnight at 4 °C. The next day, membranes were incubated with secondary antibodies (goat anti-rabbit monoclonal IgG, HRP-linked, 1:1000, Cell Signaling Technology, #7074S; horse anti-mouse IgG monoclonal, HRP-linked, 1:2000, Cell Signaling Technology, #7076S) diluted in blocking solution for 1 hour at room temperature. Signal development was performed using ECL detection reagent (Merck, #GERPN2109) according to the manufacturer’s instructions and images were acquired using the JESS Western system (Proteinsimple, Bio-Techne). For further detections, membranes were incubated in T-BST with added 0.1% NaN3 (Merck, #S2002) in T‑BST for 1 h at room temperature or Re-Blot Plus Strong Antibody Stripping solution for 15 min at room temperature (Merck, #2504). Quantification of protein bands was performed using ImageJ analysis software (release 1.53c).

**Bioinformatic analysis of data sets obtained from public resources**

RNA-seq data of preleukemia models and shRNA-mediated *ETV6::RUNX1* knockdown in REH cells was obtained from the ArrayExpress functional genomics data collection website (https://www.ebi.ac.uk/biostudies/arrayexpress) accession numbers E‑MTAB-6382^22^ and E-MTAB-1030811^23^. RNA expression data of leukemia subtypes and normal B cell developmental stages was obtained from the St. Jude PeCan Data Portal (https://pecan.stjude.cloud)^24,25^ and from the R2 Genomics Analysis Visualization Platform (http://r2.amc.nl; GSE87070 dataset^26^, microarray platform u133p2; GSE24759 dataset^27^, microarray platform u133a; MILE study, GSE13159 dataset^28^, microarray platform u133p2). Processed DNA methylation (Infinium HumanMethylation450 BeadChip platform) and matched RNA expression data (microarray platform u133p2) of various leukemia entities was retrieved from the Gene Expression Omnibus database (NCBI GEO, https://www.ncbi.nlm.nih.gov/geo) accession number GSE49032^29^. Analysis of RNA expression data was performed as described for bulk RNA-seq using the Partek Flow software. RNA-seq data of normal B cell developmental stages and *ETV6::RUNX1*+ BCP‑ALL samples was retrieved from NCBI GEO (accession number GSE115656^30^) and processed using the Galaxy platform (https://usegalaxy.eu). Fastq files were trimmed using the Trimmomatic tool and aligned to the hg38 genome using the HISAT2 aligner. Expression was quantified using htseq-count against the UCSC database. To exclude effects of underlying predisposing syndromes, one *ETV6::RUNX1*+ BCP-ALL patient presenting with trisomy 21 was omitted from the analysis. Analysis of scRNA-seq data derived from normal bone marrow precursor B cells was performed as previously described^31^. Fetal liver scRNA-seq data was derived from the Developmental Cell Atlas accession number E-MTAB-7407^32^ (Newcastle University, https://www.humancellatlas.org/). ChIP-seq datasets of REH cells for H3K4me1, H3K4me3, H3K27ac and RUNX1 (accession number GSE117684^33^), as well as ETV6::RUNX1 (accession number GSE176084^34^) were downloaded from NCBI GEO. Fastq files were processed as described for bulk RNA-seq using the Partek Flow software and BAM files were visualized using IGV version 2.9.1^35^ (https://igv.org).

**Supplementary References**

1. Vogt M, Dienstbier N, Schliehe-Diecks J, et al. Co-targeting HSP90 alpha and CDK7 overcomes resistance against HSP90 inhibitors in BCR-ABL1+ leukemia cells. *Cell Death & Disease*. Dec 6 2023;14(12):799. doi:10.1038/s41419-023-06337-3

2. Panina Y, Germond A, Masui S, Watanabe TM. Validation of Common Housekeeping Genes as Reference for qPCR Gene Expression Analysis During iPS Reprogramming Process. *Sci Rep*. Jun 7 2018;8(1):8716. doi:10.1038/s41598-018-26707-8

3. Taylor SC, Nadeau K, Abbasi M, Lachance C, Nguyen M, Fenrich J. The Ultimate qPCR Experiment: Producing Publication Quality, Reproducible Data the First Time. *Trends Biotechnol*. Jul 2019;37(7):761-774. doi:10.1016/j.tibtech.2018.12.002

4. Pellin D, Loperfido M, Baricordi C, et al. A comprehensive single cell transcriptional landscape of human hematopoietic progenitors. *Nature Communications*. 2019/06/03 2019;10(1):2395. doi:10.1038/s41467-019-10291-0

5. Riccardi C, Nicoletti I. Analysis of apoptosis by propidium iodide staining and flow cytometry. *Nat Protoc*. 2006;1(3):1458-61. doi:10.1038/nprot.2006.238

6. Xing Y, Yu T, Wu YN, Roy M, Kim J, Lee C. An expectation-maximization algorithm for probabilistic reconstructions of full-length isoforms from splice graphs. *Nucleic Acids Research*. 2006;34(10):3150-3160. doi:10.1093/nar/gkl396

7. Ewels PA, Peltzer A, Fillinger S, et al. The nf-core framework for community-curated bioinformatics pipelines. *Nature Biotechnology*. 2020/03/01 2020;38(3):276-278. doi:10.1038/s41587-020-0439-x

8. Krämer A, Green J, Pollard J, Jr, Tugendreich S. Causal analysis approaches in Ingenuity Pathway Analysis. *Bioinformatics*. 2013;30(4):523-530. doi:10.1093/bioinformatics/btt703

9. Shannon P, Markiel A, Ozier O, et al. Cytoscape: a software environment for integrated models of biomolecular interaction networks. *Genome Research*. Nov 2003;13(11):2498-2504. doi:10.1101/gr.1239303

10. Reimand J, Isserlin R, Voisin V, et al. Pathway enrichment analysis and visualization of omics data using g:Profiler, GSEA, Cytoscape and EnrichmentMap. *Nature Protocols*. Feb 2019;14(2):482-517. doi:10.1038/s41596-018-0103-9

11. Fuka G, Kauer M, Kofler R, Haas OA, Panzer-Grumayer R. The leukemia-specific fusion gene ETV6/RUNX1 perturbs distinct key biological functions primarily by gene repression. *PLoS One*. 2011;6(10):26348. doi:10.1371/journal.pone.0026348

12. Osorio D, Cai JJ. Systematic determination of the mitochondrial proportion in human and mice tissues for single-cell RNA-sequencing data quality control. *Bioinformatics*. 2020;37(7):963-967. doi:10.1093/bioinformatics/btaa751

13. Ranek JS, Stallaert W, Milner JJ, et al. DELVE: feature selection for preserving biological trajectories in single-cell data. *Nature Communications*. 2024/03/29 2024;15(1):2765. doi:10.1038/s41467-024-46773-z

14. Tirosh I, Izar B, Prakadan SM, et al. Dissecting the multicellular ecosystem of metastatic melanoma by single-cell RNA-seq. *Science*. 2016;352(6282):189-196. doi:doi:10.1126/science.aad0501

15. Phipson B, Sim CB, Porrello ER, Hewitt AW, Powell J, Oshlack A. propeller: testing for differences in cell type proportions in single cell data. *Bioinformatics*. Oct 14 2022;38(20):4720-4726. doi:10.1093/bioinformatics/btac582

16. Simmons S. Cell Type Composition Analysis: Comparison of statistical methods. *bioRxiv*. 2022:2022.02.04.479123. doi:10.1101/2022.02.04.479123

17. Jardine L, Webb S, Goh I, et al. Blood and immune development in human fetal bone marrow and Down syndrome. *Nature*. 2021/10/01 2021;598(7880):327-331. doi:10.1038/s41586-021-03929-x

18. Hao Y, Stuart T, Kowalski MH, et al. Dictionary learning for integrative, multimodal and scalable single-cell analysis. *Nature Biotechnology*. 2024/02/01 2024;42(2):293-304. doi:10.1038/s41587-023-01767-y

19. Zhang X, Song B, Carlino MJ, et al. An immunophenotype-coupled transcriptomic atlas of human hematopoietic progenitors. *Nature Immunology*. 2024/04/01 2024;25(4):703-715. doi:10.1038/s41590-024-01782-4

20. DePasquale EAK, Schnell D, Dexheimer P, et al. cellHarmony: cell-level matching and holistic comparison of single-cell transcriptomes. *Nucleic Acids Research*. 2019;47(21):e138-e138. doi:10.1093/nar/gkz789

21. Chen Y, Chen L, Lun ATL, Baldoni PL, Smyth GK. edgeR v4: powerful differential analysis of sequencing data with expanded functionality and improved support for small counts and larger datasets. *bioRxiv*. 2024:2024.01.21.576131. doi:10.1101/2024.01.21.576131

22. Böiers C, Richardson SE, Laycock E, et al. A human iPS model implicates embryonic B-myeloid fate restriction as developmental susceptibility to B acute lymphoblastic leukemia-associated ETV6-RUNX1. *Developmental Cell*. Feb 5 2018;44(3):362-377. doi:10.1016/j.devcel.2017.12.005

23. Wray JP, Deltcheva EM, Boiers C, et al. Regulome analysis in B-acute lymphoblastic leukemia exposes Core Binding Factor addiction as a therapeutic vulnerability. *Nature Communications*. 2022/11/21 2022;13(1):7124. doi:10.1038/s41467-022-34653-3

24. Downing JR, Wilson RK, Zhang J, et al. The Pediatric Cancer Genome Project. *Nature Genetics*. May 29 2012;44(6):619-622. doi:10.1038/ng.2287

25. McLeod C, Gout AM, Zhou X, et al. St. Jude Cloud: A Pediatric Cancer Genomic Data-Sharing Ecosystem. *Cancer Discovery*. 2021;11(5):1082-1099. doi:10.1158/2159-8290.Cd-20-1230

26. Polak R, Bierings MB, van der Leije CS, et al. Autophagy inhibition as a potential future targeted therapy for ETV6-RUNX1-driven B-cell precursor acute lymphoblastic leukemia. *Haematologica*. Apr 2019;104(4):738-748. doi:10.3324/haematol.2018.193631

27. Novershtern N, Subramanian A, Lawton LN, et al. Densely interconnected transcriptional circuits control cell states in human hematopoiesis. *Cell*. Jan 21 2011;144(2):296-309. doi:10.1016/j.cell.2011.01.004

28. Kohlmann A, Kipps TJ, Rassenti LZ, et al. An international standardization programme towards the application of gene expression profiling in routine leukaemia diagnostics: the Microarray Innovations in LEukemia study prephase. *British Journal of Haematology*. Sep 2008;142(5):802-807. doi:10.1111/j.1365-2141.2008.07261.x

29. Nordlund J, Bäcklin CL, Wahlberg P, et al. Genome-wide signatures of differential DNA methylation in pediatric acute lymphoblastic leukemia. *Genome Biology*. 2013/09/24 2013;14(9):105. doi:10.1186/gb-2013-14-9-r105

30. Black KL, Naqvi AS, Asnani M, et al. Aberrant splicing in B-cell acute lymphoblastic leukemia. *Nucleic Acids Res*. Nov 30 2018;46(21):11357-11369. doi:10.1093/nar/gky946

31. Mehtonen J, Teppo S, Lahnalampi M, et al. Single cell characterization of B-lymphoid differentiation and leukemic cell states during chemotherapy in ETV6-RUNX1-positive pediatric leukemia identifies drug-targetable transcription factor activities. *Genome Medicine*. Nov 20 2020;12(1):99. doi:10.1186/s13073-020-00799-2

32. Popescu D-M, Botting RA, Stephenson E, et al. Decoding human fetal liver haematopoiesis. *Nature*. 2019/10// 2019;574(7778):365-371. doi:10.1038/s41586-019-1652-y

33. Jakobczyk H, Debaize L, Soubise B, et al. Reduction of RUNX1 transcription factor activity by a CBFA2T3-mimicking peptide: application to B cell precursor acute lymphoblastic leukemia. *Journal of Hematology & Oncology*. Mar 20 2021;14(1):47. doi:10.1186/s13045-021-01051-z

34. Jakobczyk H, Jiang Y, Debaize L, et al. ETV6-RUNX1 and RUNX1 directly regulate RAG1 expression: one more step in the understanding of childhood B-cell acute lymphoblastic leukemia leukemogenesis. *Leukemia*. 2022/02/01 2022;36(2):549-554. doi:10.1038/s41375-021-01409-9

35. Robinson JT, Thorvaldsdottir H, Winckler W, et al. Integrative genomics viewer. *Nature Biotechnology*. Jan 2011;29(1):24-26. doi:10.1038/nbt.1754

**Supplementary Figures**

**
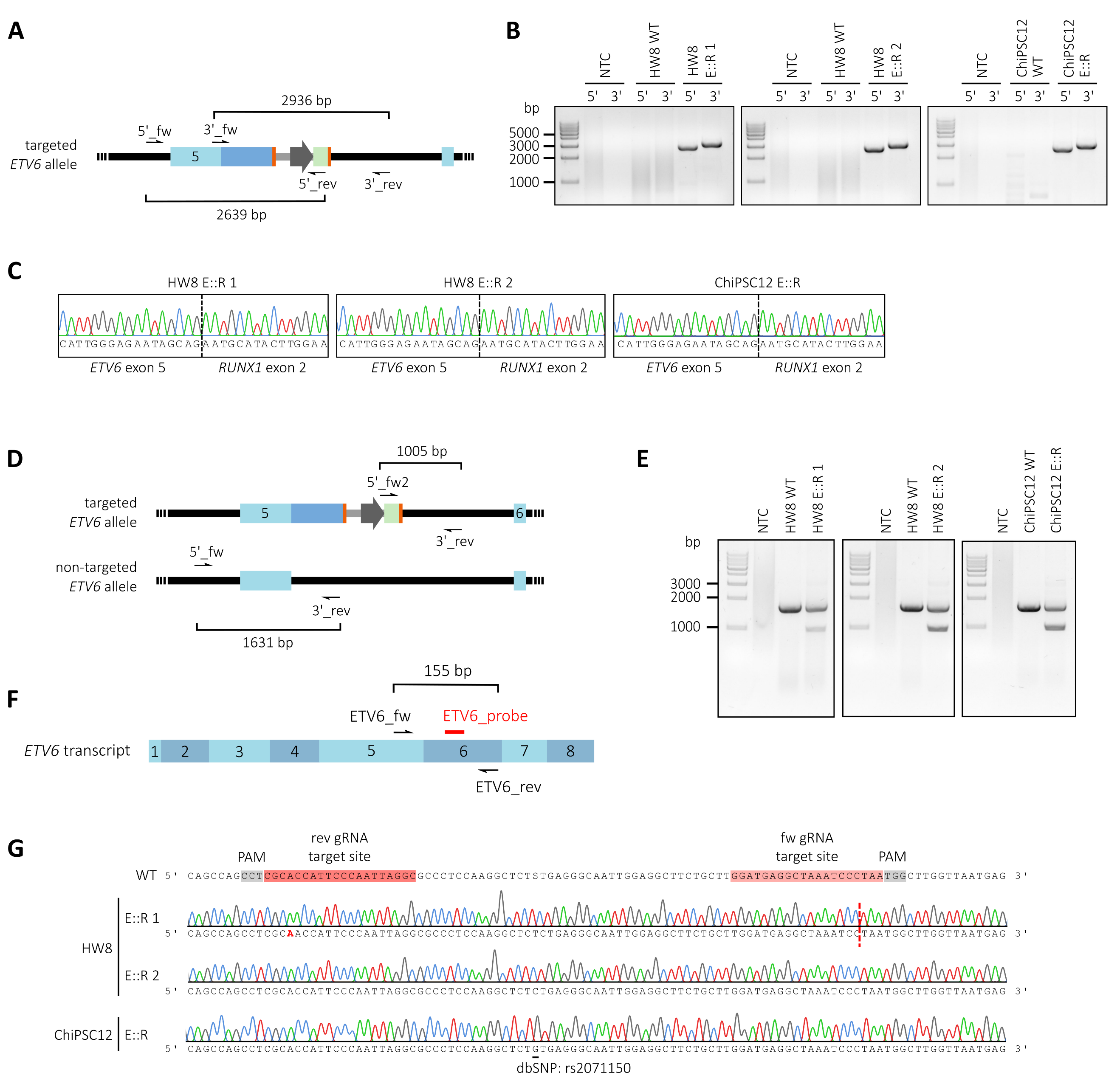
**

**Figure S1. CRISPR/Cas9-edited HW8 and ChiPSC12 hiPSCs stably express *ETV6::RUNX1* from the endogenous *ETV6* locus.** **(A)** Schematic representation of genotyping PCRs confirming correct insertion of the *RUNX1* HDR template into the *ETV6* locus of HW8 and ChiPSC12 hiPSCs (5’_PCR=2639 bp, 3’_PCR=2936 bp). Arrows indicate binding sites of PCR primers. **(B)**Genotyping PCRs confirming correct insertion of the *RUNX1* HDR template into the *ETV6* locus of HW8 and ChiPSC12 hiPSCs. **(C)** Sanger sequencing tracks of *ETV6::RUNX1*+ hiPSC lines detecting the fusion sequence between *ETV6* exon 5 and *RUNX1* exon 2. **(D)** Schematic representation of the PCR approach used for detection of *ETV6::RUNX1* homo- or heterozygosity in hiPSCs (WT allele=1631 bp, targeted allele=1005 bp). **(E)** PCRs confirming heterozygous allele status of *ETV6::RUNX1* in HW8 and ChiPSC12 hiPSCs. **(F)** Schematic representation of the *ETV6* RT-qPCR design. The RT-qPCR probe sequence is marked in red. NTC: no-template control (nuclease-free H_2_O). **(G)** Sanger sequencing of the gRNA binding region within *ETV6* intron 5 in the three *ETV6::RUNX1*+ hiPSC clones.


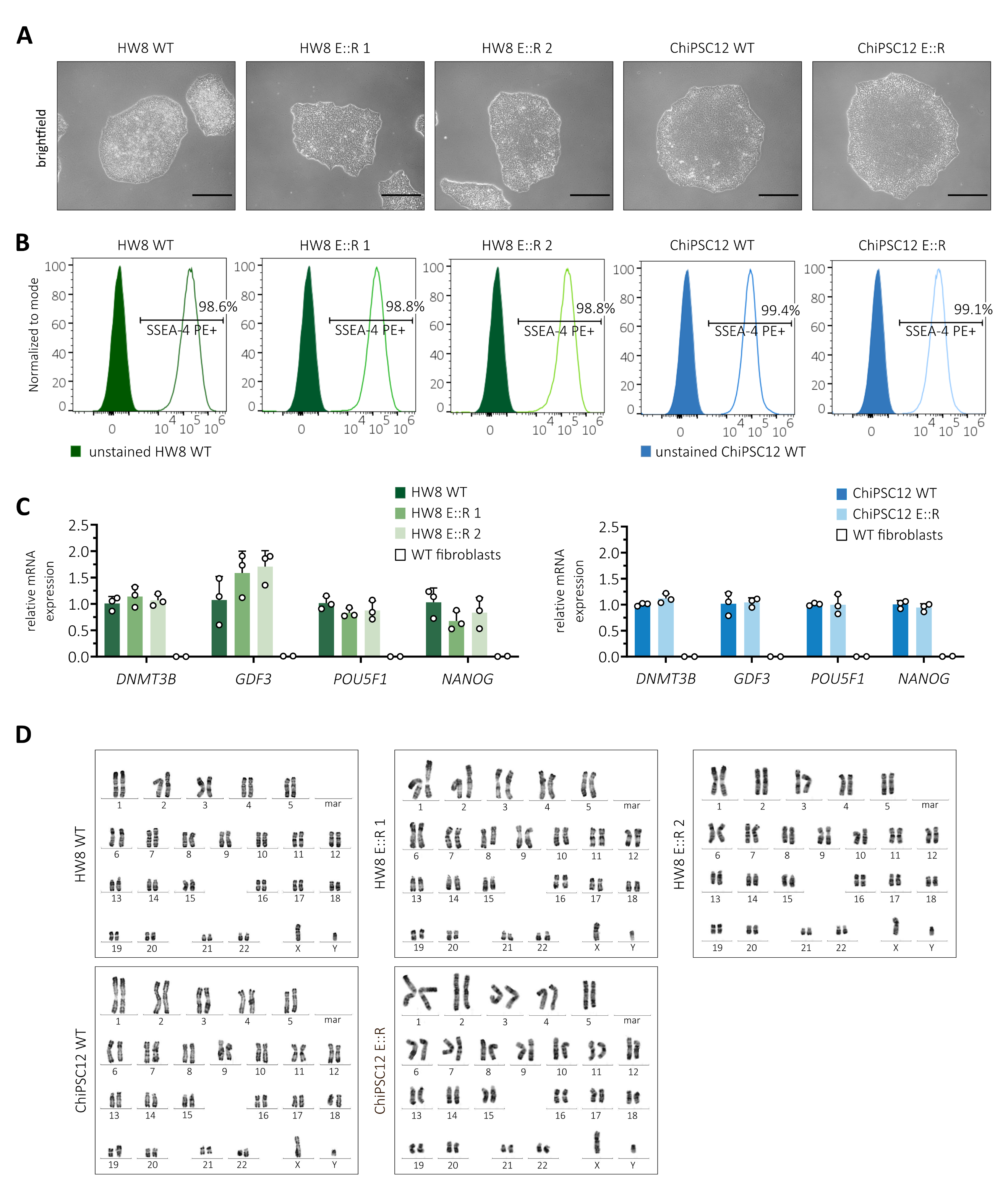


**Figure S2. Assessment of hiPSC quality. (A)** Representative brightfield images confirming normal hiPSC colony morphology (scale bar=300 µm). **(B)** Flow cytometric analysis of stage‑specific embryonic antigen 4 (SSEA-4) on HW8 WT and ChiPSC12 WT cells, as well as CRISPR/Cas9-edited *ETV6::RUNX1*+ hiPSC lines HW8 E::R 1, HW8 E::R 2 and ChiPSC12 E::R. **(C)** Representative RT-qPCR analyses of pluripotency marker genes *DNMT3B*, *GDF3*, *POU5F1* and *NANOG* in HW8 WT, ChiPSC12 WT, as well as the respective ETV6::RUNX1+ hiPSC clones. Fibroblasts from two healthy donors were used as negative controls. Expression of *DNMT3B* and *GDF3* is normalized to *ATP5PB*, expression of *POU5F1* and *NANOG* is normalized to *PGK1*. Data is presented as the mean + standard deviation, and expression of *ETV6::RUNX1*+ hiPSC lines is presented relative to the respective WT. **(D)** Representative karyotype data for *ETV6::RUNX1*+ and WT hiPSCs. Karyotyping was performed by Judith Bartel at the Institute of Human Genetics (Hannover Medical School (MHH)).

**
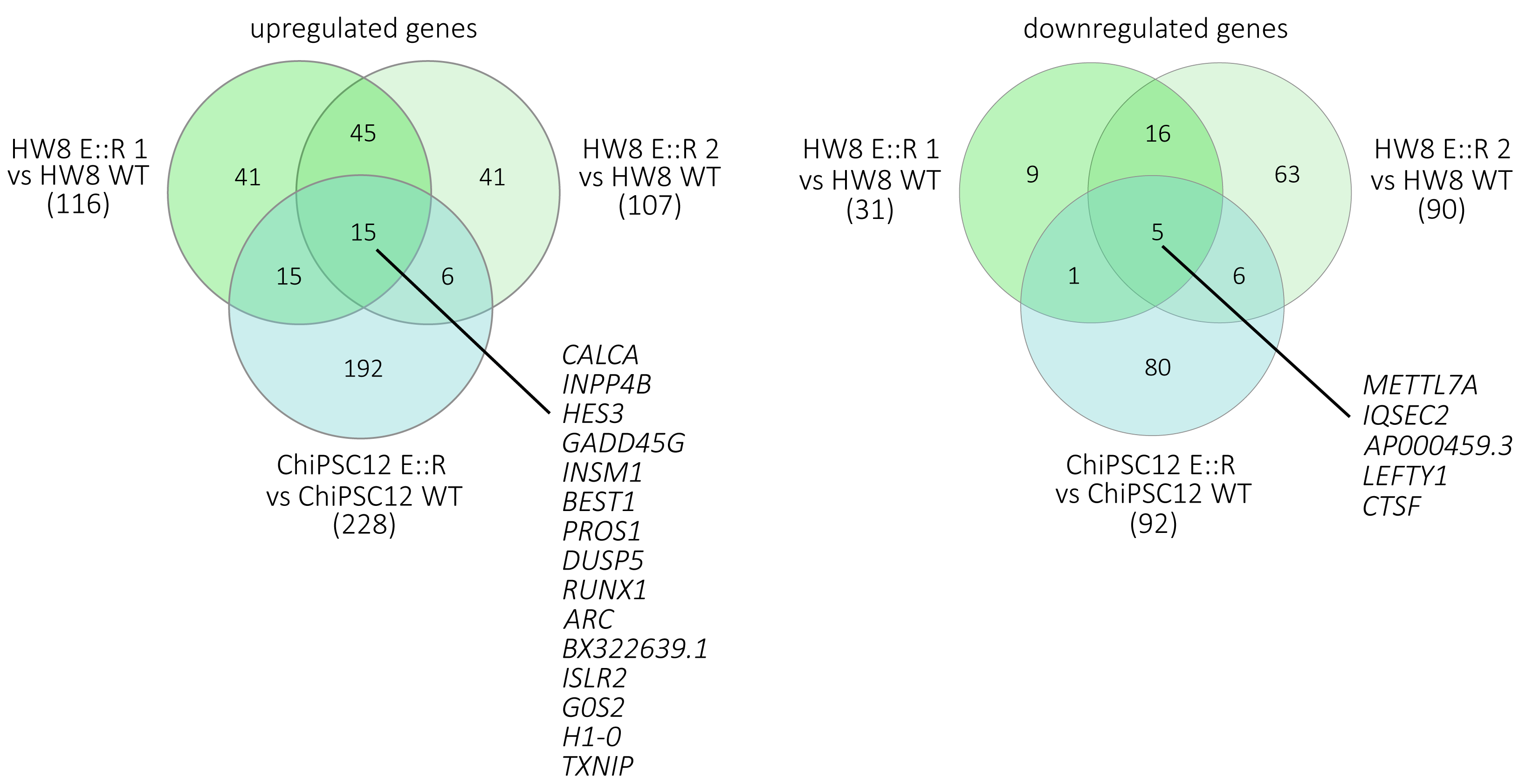
**

**Figure S3. Transcriptome analysis of *ETV6::RUNX1*+ hiPSCs.** Venn diagrams of upregulated and downregulated differentially expressed genes (absolute fold change >2 and p<0.05 detected in *ETV6::RUNX1*+ hiPSCs by RNA-seq compared to their respective WT counterpart.


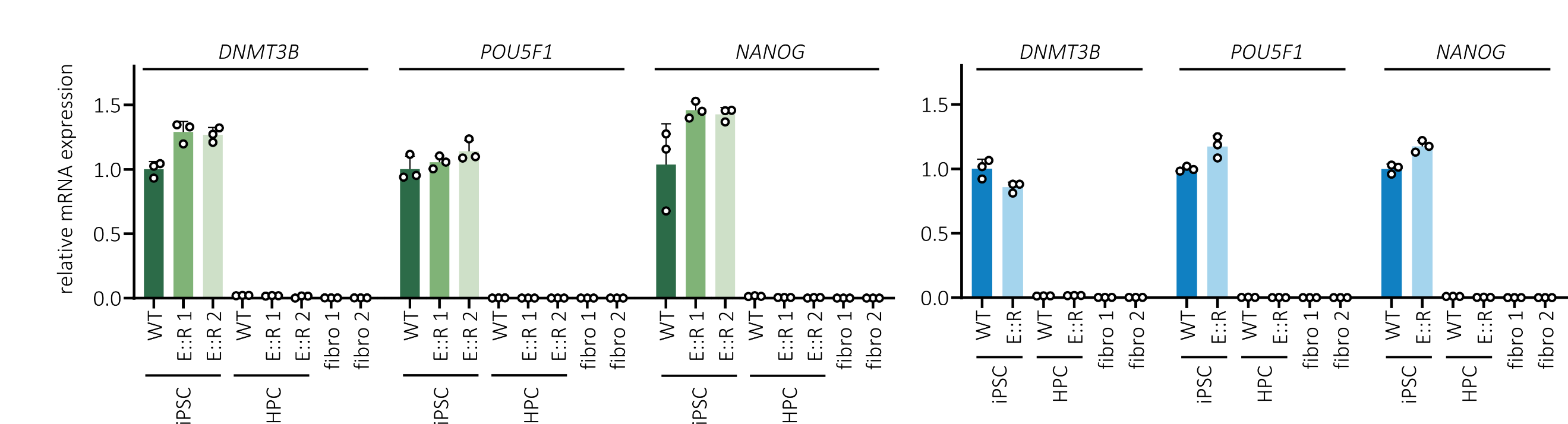


**Figure S4. Pluripotency marker gene expression HPCs.** RT‑qPCR analysis of pluripotency marker genes *DNMT3B*, *POU5F1* (*OCT4*) and *NANOG* in *ETV6::RUNX1*+ and WT HPCs and hiPSC clones. Fibroblasts from two healthy donors were used as negative controls. Expression of *DNMT3B* is normalized *to ATP5PB*, expression of *POU5F1* and *NANOG* is normalized to *PGK1*. Data is presented as the mean + standard deviation, and normalized to the respective WT hiPSCs.

**
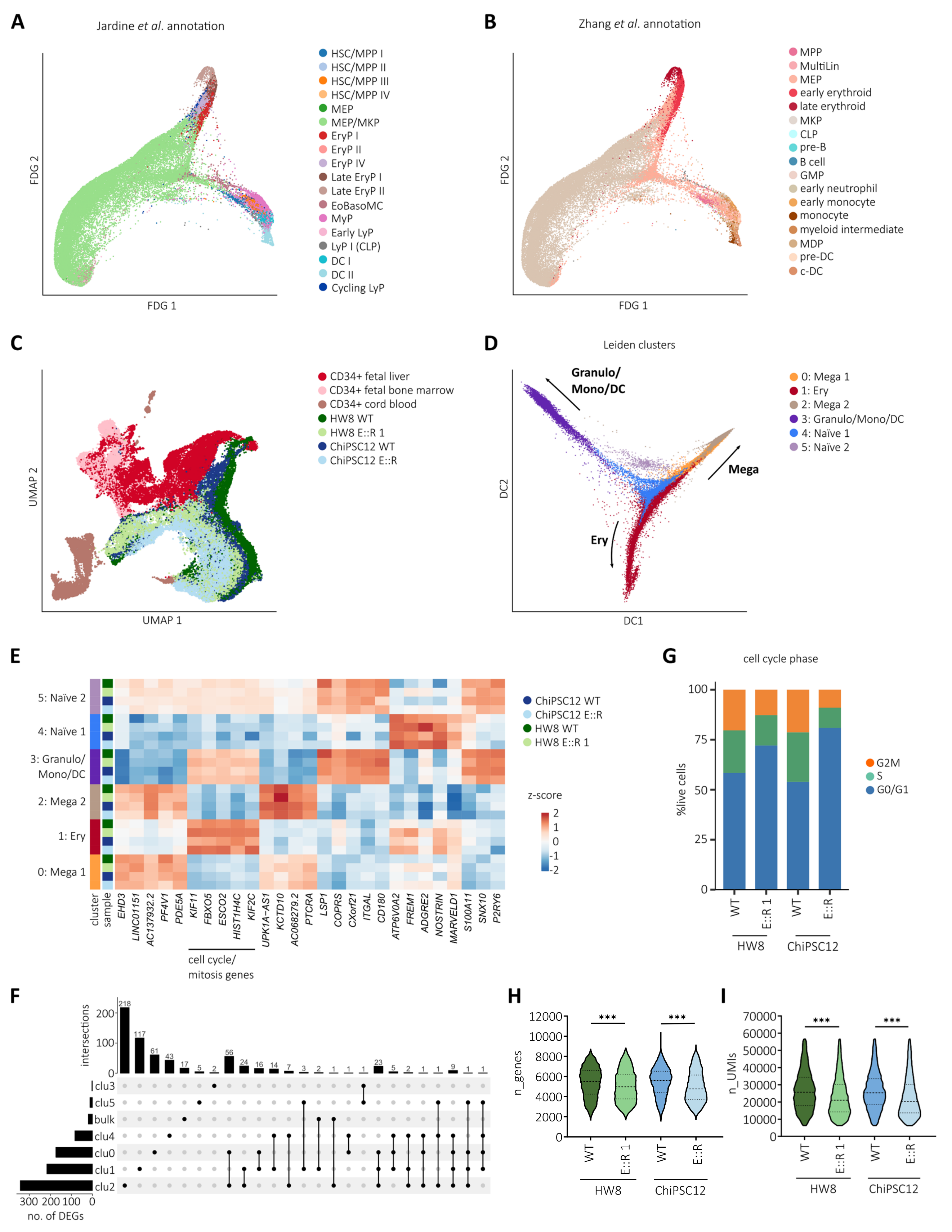
**

**Figure S5. scRNA-seq analysis of hiPSC-derived HPCs. (A-B)** Force-directed graph (FDG) plots of hiPSC-derived HPCs annotated using **(A)** published fetal bone marrow scRNA-seq data enriched for CD34+ cells^17^ and **(B)** published adult bone marrow scRNA-seq data^19^. **(C)** Uniform manifold approximation and projection (UMAP) plot of hiPSC-derived HPCs and published human scRNA-seq atlas data of CD34+ fetal liver, fetal bone marrow and cord blood^17^. **(D)** Diffusion plot of differentiation directories indicated by arrows. **(E)** Heat map showing expression of the top 5 upregulated marker genes for each Leiden cluster. **(F)** Upset plot depicting unique or shared differentially expressed genes (DEGs) in *ETV6::RUNX1*+ versus wild-type cells among scRNA-seq clusters and hiPSC bulk RNA-seq data. **(G)** Stacked bar plot showing distribution of cell cycle stages of wild-type and *ETV6::RUNX1*+ HPCs. **(H-I)** Violin plots depicting number of expressed genes (n_genes) and unique molecular identifier counts (n_UMIs) per cell detected by scRNA-seq. Median expression and quartiles are indicated. Data was analyzed for statistical significance using an unpaired t-test (***p<0.001).


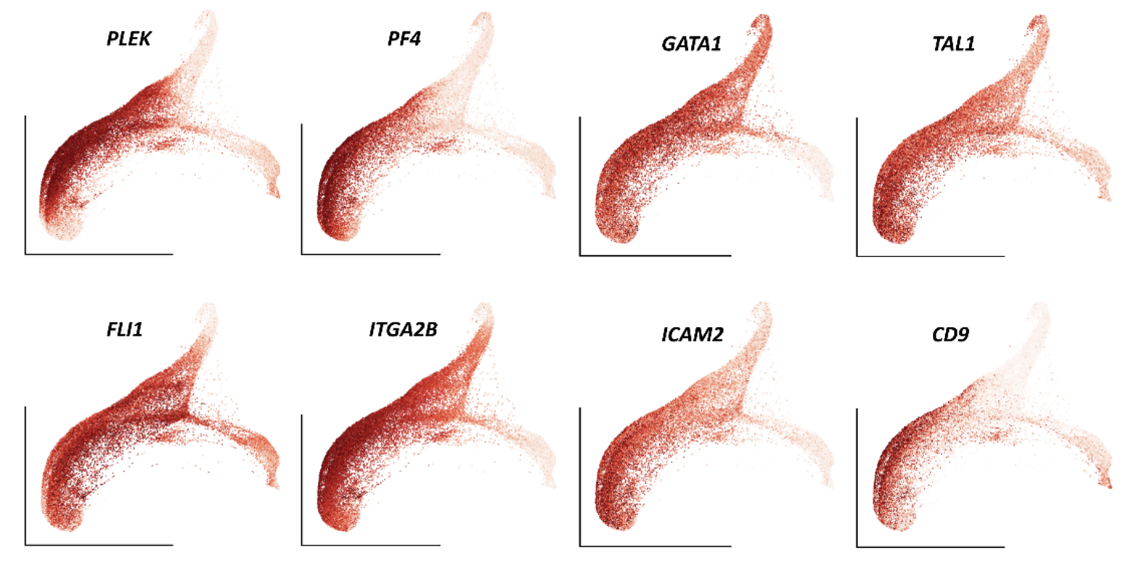


**Figure S6. Single-cell transcriptome analysis depicting megakaryocyte marker gene expression.**

**
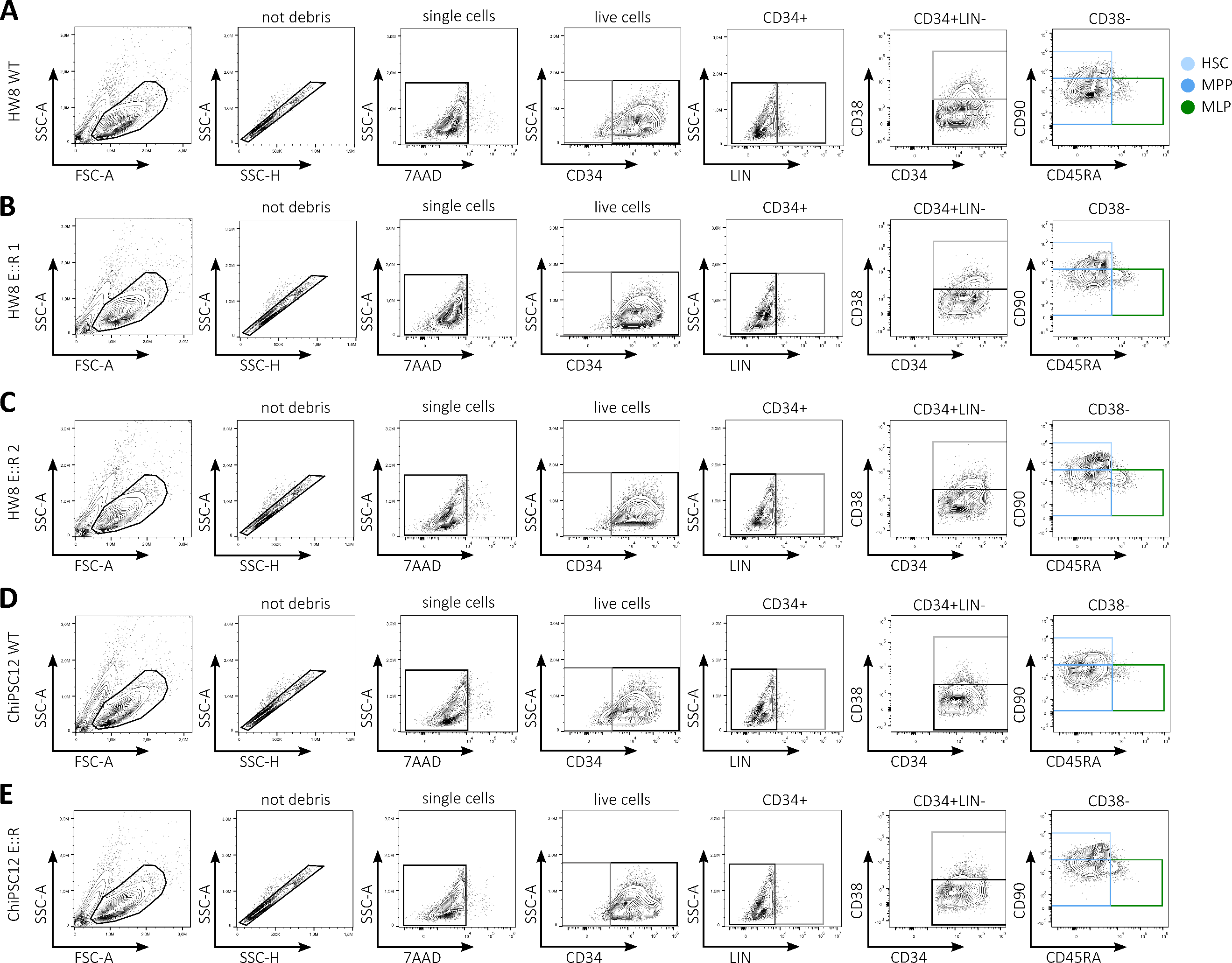
**

**Figure S7. Flow cytometric analysis of hiPSC-derived HPCs.** Gating strategy adapted from Pellin *et al*.^4^. HPCs derived from **(A)** HW8 WT, **(B)** HW8 E::R 1, **(C)** HW8 E::R 2, **(D)** ChiPSC12 WT and **(E)** ChiPSC12 E::R hiPSCs. HSC: hematopoietic stem cells, MPP: multipotent progenitors, MLP: multi-lymphoid progenitors.


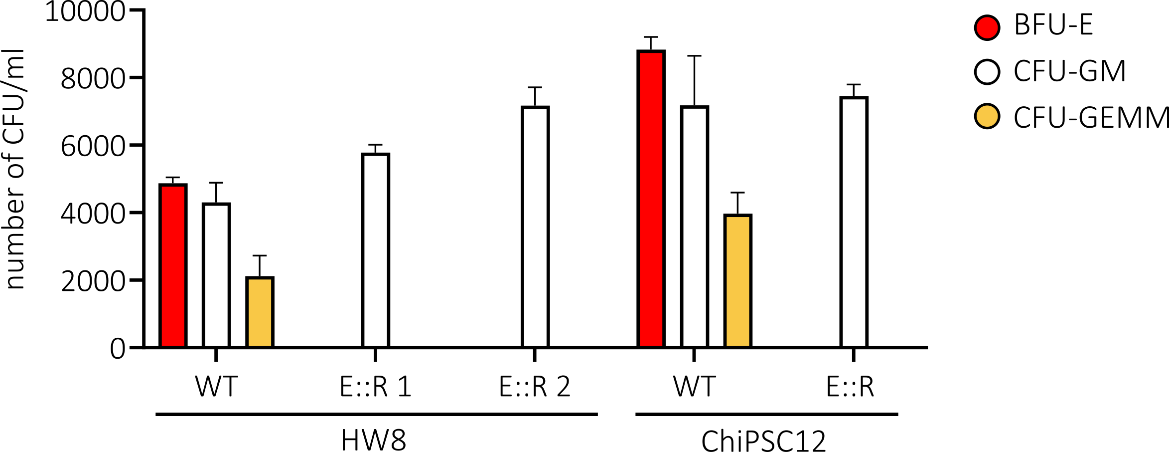


**Figure S8. Differentiation potential of HPCs.** Colony forming unit (CFU) potential of HPCs derived from WT and *ETV6::RUNX1*+ hiPSCs. Representative results of three independent experiments are shown. Mean number of CFU/ml + SEM is indicated.

**
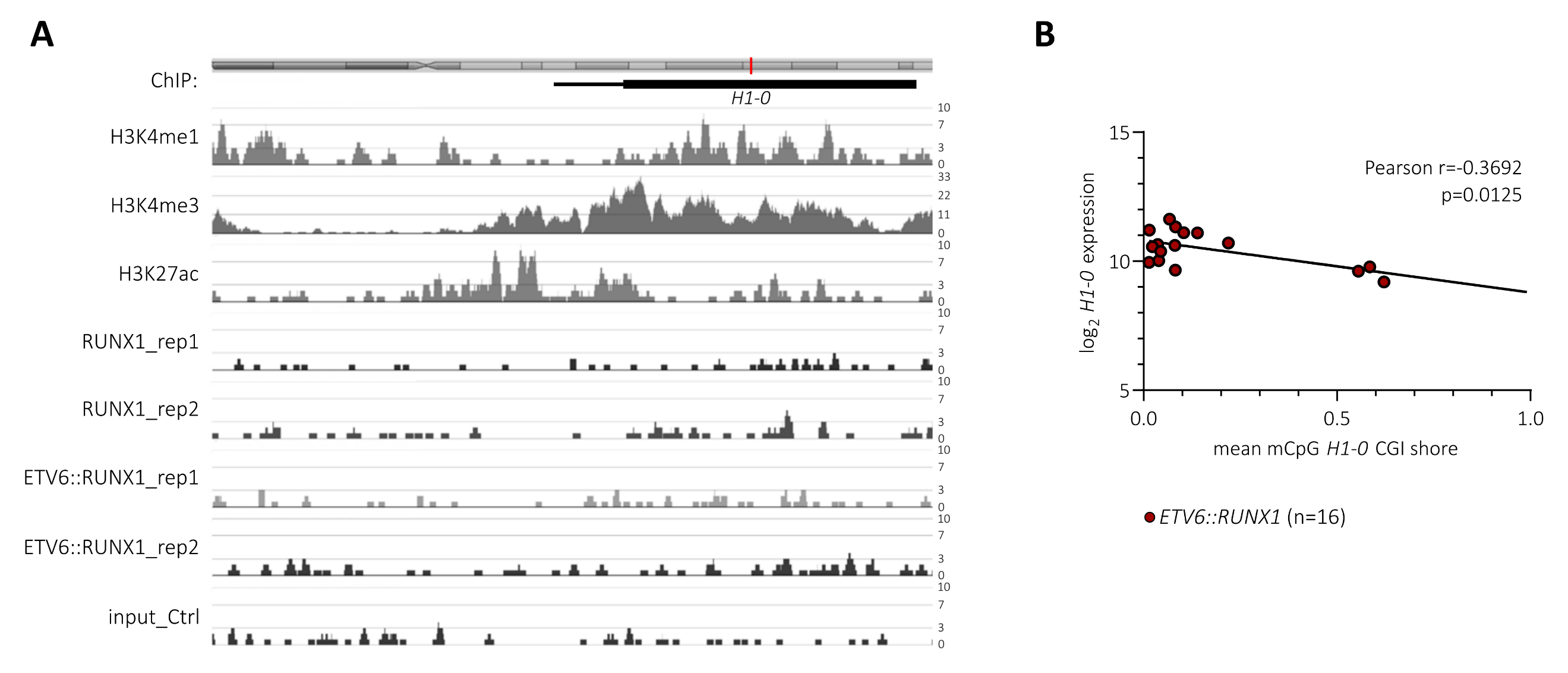
Figure S9. Chromatin immunoprecipitation (ChIP) analysis of the *H1-0* promoter region. (A)**ChIP peak visualization of the human *H1-0* gene region derived from ChIP-seq data of REH cells for H3K4me1, H3K4me3, H3K27ac and RUNX1 (accession number GSE117684^33^) as well as ETV6::RUNX1 (accession number GSE176084^34^). **(B)** Pearson correlation of *H1‑0* RNA expression and mean DNA methylation of the *H1‑0* CGI shore probes cg07141002 and cg01883777 in *ETV6::RUNX1*+ BCP-ALL patients (n=16; accession number GSE49032^29^). Expression is shown for microarray probe 208886_at. Each dot represents a single patient.

**
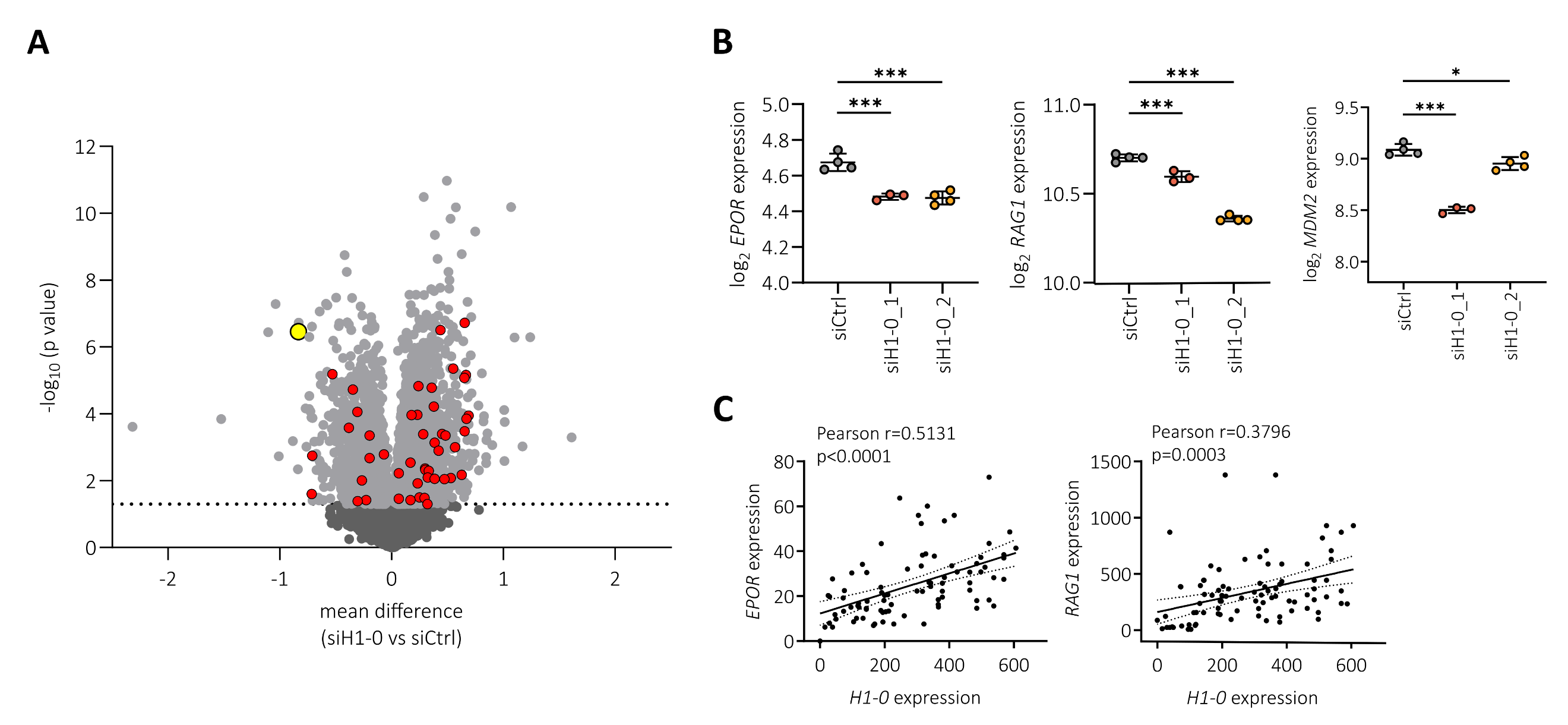
**

**Figure S10. Differential gene expression in siH1-0 versus siCtrl. (A)** Volcano plot depicting differentially expressed genes in REH cells with siRNA-mediated *H1-0* knockdown derived by RNA-seq. *H1-0* is indicated in yellow, red dots represent significant genes (p<0.05 indicated by dotted line) of the *ETV6::RUNX1* signature derived from Fuka *et al*.^11^. **(B)** RNA expression levels of *EPOR*, *RAG1* and *MDM2* determined by RNA-seq in siCtrl and siH1-0 REH. **(C)** Pearson correlation of *H1‑0* expression with *EPOR* or *RAG1* expression in *ETV6::RUNX1*+ BCP-ALL patient samples derived from the PeCan St. Jude cloud^24,25^ (n=87).


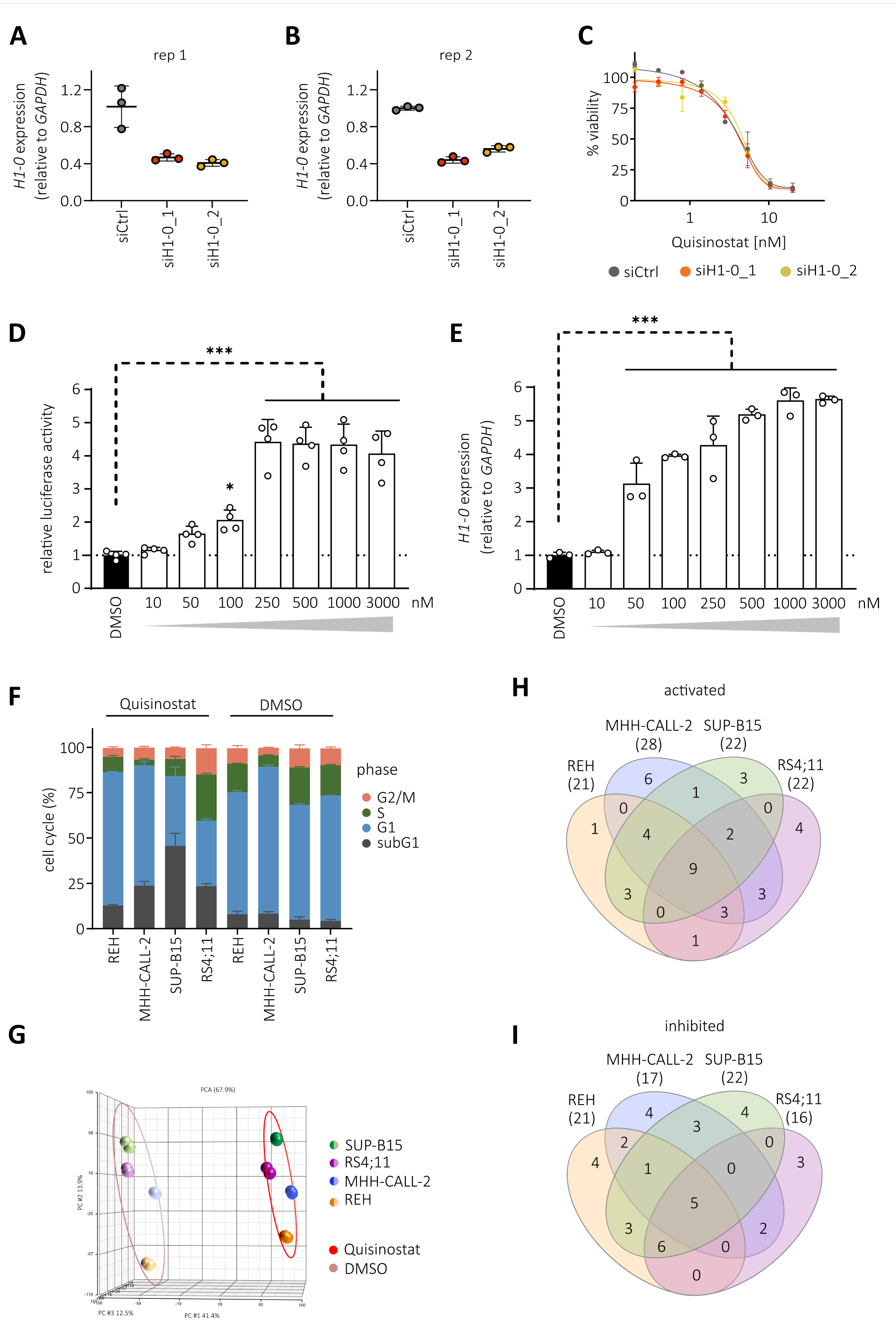


**Figure S11. Characterization of Quisinostat-induced changes. (A-B)** *H1‑0* expression determined by RT-qPCR in REH cells treated for 48 hours with a non-targeting siRNA pool (siCtrl) or *H1‑0*-targeting siRNA pools siH1-0_1 or siH1-0_2. The two biological replicates used for drug screening are shown. Data is presented as the mean ± standard deviation. **(C)** Dose-response curves of REH cells with H1-0 knockdown induced for 48h. Shown are the means ± SEM of two biological replicates of REH cells treated with Quisinostat. **(D-E)** 293T cells were transfected with vectors encoding for the *H1-0* promoter-like signature upstream of Firefly luciferase and for Renilla luciferase. **(D)** Luciferase activities were normalized to Renilla luciferase activity and the empty vector control. **(E)** *H1-0* expression was quantified by RT-qPCR and normalized to *GAPDH* levels. Data represent mean values of three independent replicates + standard deviation. Significance was calculated using an ordinary one‑way ANOVA (*p<0.05, ***p<0.001). **(F)** PCA plot of Quisinostat and DMSO-treated BCP-ALL cells. **(G)** Cell cycle distribution of BCP-ALL cells treated with 1 µM Quisinostat or DMSO for 24h determined by Nicoletti assay. Data is presented as the mean + standard deviation. **(H-I)** Venn diagrams of significantly **(H)** activated or **(I)** inhibited upstream regulators (p-value<10^-9^, absolute activation z-score>0.5) determined by Ingenuity Pathway Analysis (IPA, QIAGEN) in BCP-ALL cell lines treated with 1 µM Quisinostat compared to DMSO treatment.

**
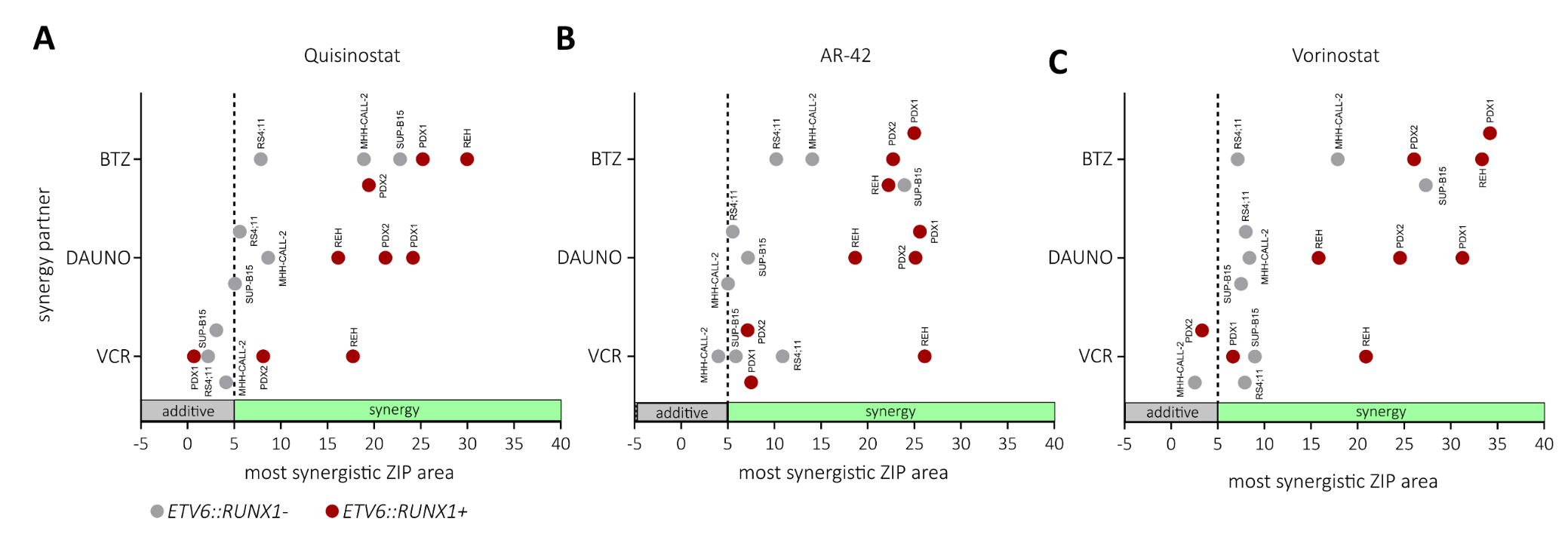
**

**Figure S12. Drug screen analyses in *ETV6::RUNX1*+ and *ETV6::RUNX1*- samples.** Mean most synergistic area scores (2 x 2 dose window) of the HDACis **(A)** Quisinostat, **(B)** AR-42 or **(C)** Vorinostat with Vincristine (VCR), Daunorubicin (DAUNO) or Bortezomib (BTZ) across four BCP-ALL cell lines and two *ETV6::RUNX1*+ PDX. Drug screens of BCP-ALL cell lines were performed as triplicates for Quisinostat and duplicates for AR-42 and Vorinostat. For PDX, screenings were performed only once due to limited amount of cells.
